# Genetic Differentiation of Reed Canarygrass (*Phalaris arundinacea* L.) within Eastern Europe and Eurasia

**DOI:** 10.3390/genes15060734

**Published:** 2024-06-03

**Authors:** Neil O. Anderson, Edvina Krokaitė-Kudakienė, Lina Jocienė, Tomas Rekašius, Olga A. Chernyagina, Algimantas Paulauskas, Eugenija Kupčinskienė

**Affiliations:** 1 Department of Horticultural Science, University of Minnesota, 1970 Folwell Ave., St. Paul, MN 55108, USA; 2 Department of Biology, Faculty of Natural Sciences, Vytautas Magnus University, K. Donelaičio Str. 58, 44248 Kaunas, Lithuania; edvina.krokaite@vdu.lt (E.K.-K.); lina.jociene@vdu.lt (L.J.); algimantas.paulauskas@vdu.lt (A.P.); e.kupcinskiene@gmail.com (E.K.); 3 Department of Mathematics and Statistics, Faculty of Informatics, Vytautas Magnus University, K. Donelaičio Str. 58, 44248 Kaunas, Lithuania; tomas.rekasius@vgtu.lt; 4 Department of Mathematical Statistics, Faculty of Fundamental Sciences, Vilnius Gediminas Technical University, Saulėtekio Ave. 11, 10223 Vilnius, Lithuania; 5 Kamchatka Branch of the Pacific Institute of Geography of the Far Eastern Branch of the Russian Academy of Sciences, Partizanskaya Str. 6, 683001 Petropavlovsk-Kamchatskyii, Russia; kamchatika@mail.ru

**Keywords:** natural distribution range, invasive plants, Poaceae, riparian species, genetic differentiation, aquatic plants, microsatellite molecular markers, single nucleotide polymorphisms

## Abstract

Worldwide molecular research of economically important *Phalaris arundinacea* (Poaceae) is mainly focused on the invasions of this species from Europe to North America. Until the present study, the genetic diversity of the *P. arundinacea* had not been studied across the Baltic countries. The objective of this research is to evaluate the diversity of Lithuanian populations of *P. arundinacea* at simple sequence repeat (SSR) loci comparatively among populations of the Baltic countries, Luxembourg, and the Russian Far East (Eurasian), evaluating differentiation between Lithuanian populations and ornamental accessions, and relating these with environmental features. For six selected Lithuanian river basin populations, GBS low density SNPs were used to determine genetic diversity. Bayesian analysis showed that Eurasian populations of *Phalaris arundinacea* consist of two gene clusters. Statistically significant genetic differentiation among European and Eurasian populations was documented. Lithuanian genotypes growing naturally along rivers are genetically distinct from cultivated ornamentals. GBS-SNPs divided the six selected Nemunas river basins into three distinct groups with one, two, or three rivers in separate groupings for genetic diversity. Genetic diversity is primarily within, rather than among, Lithuanian, eastern European, and Eurasian populations of *P. arundinacea* across the continent. Thus, restoration efforts would benefit from local population seed origination.

## 1. Introduction

Sustainable development of agriculture requires the preservation and ecological balance in the use of natural resources. Riparian plant species play a key role in aquatic ecosystems, and data on their status, habitats, and spatial and temporal changes are of particular importance [1]. In recent decades, the United Nations has paid exceptional attention to wetland ecosystems, which are very rich in biodiversity. Under the Ramsar Convention, countries around the world have committed to protecting wetlands as important habitats for animals and plants [1]. The fourth plan of the Ramsar Convention was drawn up for the period of 2016–2024 [2]. The European ecological network (Natura 2000) has also been established for similar purposes [3].

All soil and climate changes are significantly affecting the condition of groundwater and water bodies [4]. Due to the world’s human population growth, demand for quantity of freshwater is increasing. In the Fifth International Meeting of Heads of Ecology, anthropogenic impacts on biodiversity, biological invasions, ecosystem welfare, and river basin management have been identified as significant drivers in, or the products of, climate change [5]. In the temperate climate zones, one of the most common macrophyte wetland species is reed canarygrass (*Phalaris arundinacea* L.).

Reed canarygrass is a perennial geophyte, representative of *Cyperales* order, *Poaceae* family, *Pooideae* subfamily, *Poodae* supertribe, *Poeae* tribe, *Phalaridinae* subtribe, and one of 22 species in genus *Phalaris* [6,7,8]. It grows up to 2.2 m in height, flowering in June–July [9,10,11], reproducing by rhizomes, shoots, and seeds [12,13,14,15], and is a cross-pollinated, anemophilous species [16]. Together with many other forms, a striped form of *Phalaris arundinacea* var. *picta* (with the white or yellow stripes on the leaves), is worldwide in distribution [17]. *Phalaris arundinacea* is a temperate climate plant occupying open areas [18], tolerant of a wide range of precipitation [19,20] and temperatures [20,21,22,23], and grows in humid soils [24] rich in inorganic forms of nitrogen [16,18,25] with acidity ranging from moderately acidic to slightly alkaline [24]. It is classified as moderately tolerant to salt [18]. The plant forms large and lush meadows on the banks of rivers, ditches, or lakes [9,26], growing as deep as 30 cm in open water.

It is spread in the northern hemisphere [27], naturally growing in Europe, Asia, Africa, and North America. The natural distribution of *Phalaris arundinacea* in N. America has been controversial, with populations on that continent originally purported to be hybrids of local accessions and imported cultivars from Europe [28]. More recent studies in the midwestern U.S. have found all riparian populations and those along highway corridors to be native to N. America [29]. As an invasive species in U.S. wetlands, it outcompetes aggressively with native species [25,30,31,32,33,34].

It is an economically important species in Europe and the U.S., used for forage (low alkali types) [27,35,36,37,38,39,40], as protection against soil erosion [41], for ornamental purposes [42,43,44,45,46], phytoremediation [47,48,49], and biofuel production [50,51]. While there are very few, if any, active reed canarygrass breeding programs, selection of productive cultivars would require molecular research to supply data on genetic diversity within and among populations [52,53]. This species has been examined by many molecular markers including allozymes [33], inter simple sequence repeats (ISSRs), [12,17,53,54], amplified fragment length polymorphism (AFLP) [52], simple sequence repeats (SSRs) [55,56,57,58,59,60], internal transcribed spacers (ITSs) [61], chloroplast DNA (cpDNA) markers [52,55,56,60,62], and single nucleotide polymorphisms (SNPs) from DNA sequencing [29].

Former investigations of *P. arundinacea* have been primarily focused on genomic comparisons of populations among the two continents, either N. America and Europe, or N. America and Western Asia [12,29,52,53,54,55,56,57,58,60]. Most European continent studies included several populations of the countries: Norway [12,59,62], Sweden [12,55,56,59,60,62], Finland [59,60,62], Denmark [59,62], Germany [12,56,59,62], Poland [55,56,59,62], Hungary [56], the Czech Republic [54,60], Montenegro [12,59,62], Ireland [59,62], Great Britain [12,59,62], the Netherlands, Portugal [60], Switzerland [12,56,60], and the western Russian Federation [56]. In Europe, few investigations have been devoted to examining a wider range of populations within specific countries, with the exception of the Czech Republic [53] or Romanian assessments [17]. Such genomic analyses have found that even though sampling widespread areas within continents and countries, significantly greater genetic variation occurred within rather than among populations [12,29,52,53,54,55,56,57,58,60,61,62,63]. However, genomic markers have also been useful in distinguishing among populations from different geographic locations (such as individual rivers within a country [29]) or on a larger scale, e.g., among continents. Such genomic analyses led to the seminal paper [29] reporting that all riparian populations in the state of Minnesota, U.S.A., were genetically distinct from central Europe and native to N. America [29]. This was in opposition to previous theories, based on early molecular marker techniques [64], that all N. American populations were European “exotics” [28].

Extensive genomic surveys across geographic areas of *P. arundinacea* and many other native or invasive species’ ranges have importance beyond assessing genetic variability within and among populations, river basins and/or countries. The discovery that all riparian populations in Minnesota were native [29] required philosophical, managerial, and legislative changes in perception by land managers, regulators, legislators, and research from its misconceived perception as an “exotic” to a “native” despite its continuing spread as an invasive species, a difficult challenge due to the economic costs of considerable scale spent on mitigation [37,65]. Implications from the use of genomics in shifting perceptual views of native, invasive species may have value in the commencement of a similar continental-scale study of native *P. arundinacea* across Eurasia.

We have documented the genetic diversity of native *P. arundinacea* in our pilot study of Lithuanian populations of the Merkys basin [64], which has overtaken the Baltic countries as an invasive. *Phalaris arundinacea* grows naturally and is widespread in Lithuania [11,26,65,66,67]. Further selection process is ongoing with the purpose to create varieties for production of raw material relevant for biofuel [68,69]. In the present study, focusing on reed canarygrass genetic diversity across eastern Europe, we hypothesize that Lithuanian *Phalaris arundinacea*, as a part of populations within the natural distribution range of the species, might differ in genetic diversity, in relation to environmental factors. The aim of the research is to evaluate the diversity in microsatellite markers (SSR loci) or GBS-SNPs (select populations) of Lithuanian populations of *P. arundinacea* by (a) comparing it to populations from the other Baltic countries, Luxembourg, and the Russian Far East, (b) incorporating Lithuanian genotypes cultivated for ornamental purposes, and (c) relating the genetic parameters of the riparian plant in river basins varying in river size, river pollution by nitrogen, and land cover and use class in the present state of rivers and riverbed origin.

## 2. Results

### 2.1. Genetic Diversity of Populations of P. arundinacea

For all populations, the total number of alleles per separate SSR marker ranged from 3 to 13 (Table 1). The selected markers generated 95 alleles, ranging in size from 90 to 163 bp. For all investigated populations of *P. arundinacea* of Lithuania and other countries, the average number of alleles per marker ranged from 1.2 to 6.4. For Lithuanian populations, all alleles were polymorphic.

Among all the studied Eurasian populations of *P. arundinacea*, the Lok1 (Nemunas basin) population had the lowest (18.1%) polymorphism while the Jie1 (Nemunas basin) population had the highest (40.4%) (Table S1).

Some populations of the Nemunas basin (Mer3, Nem2) and the Russian Far East (Kir1) were characterized by unique alleles. The average polymorphism among the studied population groups separated into countries was as follows: Russian Far East (25.0%) < Lithuania (29.5%), Baltic countries (30.1%), Luxembourg (33.0%; Table 2). For all European populations, the mean polymorphism (30.0%) was not significantly higher in Eurasian populations (25%) or populations of the Baltic States (30.1%) (Table 2).

### 2.2. Genetic Differentiation of Populations of P. arundinacea

**Figure 1:**
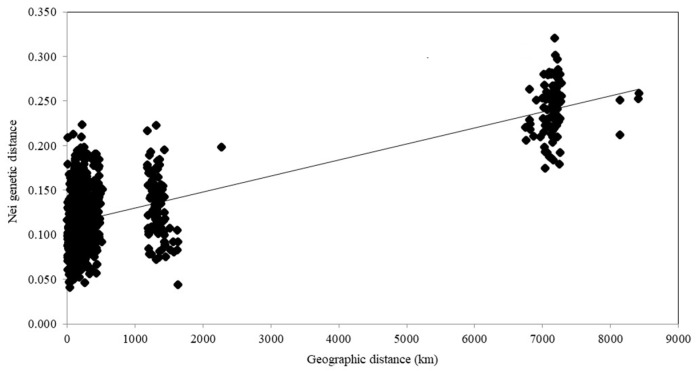
Mantel test defined for 51 Eurasian populations of *Phalaris arundinacea* among geographic distances (km) and Nei’s genetic distances at 14 microsatellite loci. The linear regression equation is y = 2 × 10^−5^x + 0.1116; R^2^ = 0.5526.

Among all of the studied Eurasian populations of *P. arundinacea*, the largest Nei’s genetic distance (0.320) was found between Nem3 (Lithuania) and Kir1 (Russian Far East) (Table S2; Figure 1). The Nei’s genetic distances among Lithuanian populations of *P. arundinacea* ranged from 0.041 to 0.224: among all the populations, both genetically the closest (Nem4 and Ses1) and the most distant (Mer2 and Mus1) (Table S2). Mantel tests showed statistically significant relations between geographic and genetic distances of the Eurasian populations of *P. arundinacea* (r = 0.743, *p* < 0.01).

**Figure 2:**
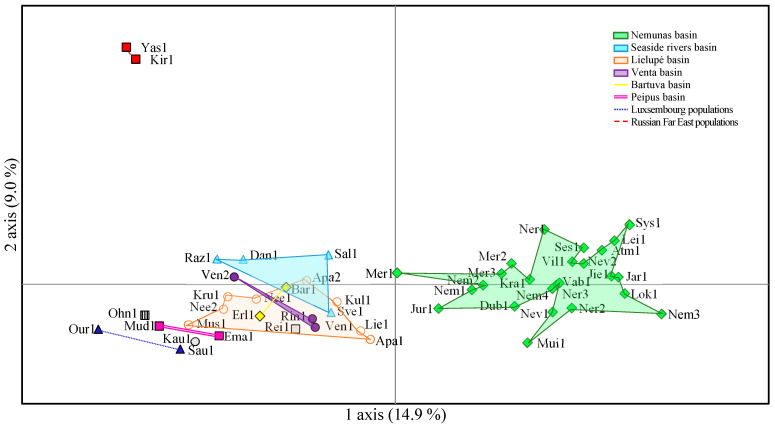
Principal coordinate analysis (1st and 2nd axis) based on microsatellite markers of *Phalaris arundinacea*, for of 51 populations: the Lithuanian basin populations (green diamonds), Eurasian (blue triangles, orange circles, fuchsia squares and purple circles) and the Russian Federation (red squares).

Based on the principal coordinate analysis (PCA) which accounts for 23.9% of the genetic variation, the populations were divided geographically (Figure 2). The most distinguished were Russian Far Eastern populations (red color; Figure 2) and Nemunas. The 1st principal coordinate explains 14.9% of the total diversity, separating the populations of Nemunas basin from all other populations. The 2nd principal coordinate explains 9.0% of the diversity, separating the populations of the Russian Far East from all other populations of Europe. The remaining populations across the European continent were closer to each other, but slightly separated by river basins. In the principal coordinate analysis plot, the populations of the Coastal rivers basin and the populations of Luxembourg were more separate from the other populations; the populations of the Venta basin were allocated together with the populations of the Bartuva and Lielupe basins (Figure 2).

Very highly statistically significant genetic differentiation by hierarchical AMOVAs was revealed among population groups (*Φ_CT_*) of the Nemunas and all other Lithuanian river basins (Lielupe, Bartuva, Venta) tested as well as among populations within groups (*Φ_SC_*), and within populations (*Φ_ST_*; Table 3). The populations of Nemunas and Bartuva river basins were the most differentiated, and the populations of the Nemunas and Venta river basin were the least. Within populations, differentiation (*Φ_SC_*) was the highest and ranged from 0.176 to 0.236.

Genetic differentiation by hierarchical AMOVA among population groups of *P. arundinacea* (*Φ_SC_*) according to land cover and use type was small, ranging from *Φ_SC_* = 0.169 to *Φ_SC_* = 0.80, but were all statistically significant (Table 4). The division of populations into groups by present river status also revealed significant genetic differentiation. Within population groups (*Φ_ST_*), based on regions of nitrogen pollution of the rivers in year 1992–1996, had statistically significant genetic differentiation. The division of populations into groups depending on whether they are beside different land cover types, river states, rivers sizes, and riverbed origins (*Φ_CT_*) also had significant levels of genetic differentiation among and within Lithuanian populations; within population differences were the highest at 82% (Table 4).

Grouping of Eurasian populations of *P. arundinacea* by continents (Europe and Asia) showed very highly statistically significant genetic differentiation (Table 5). The division of European populations into groups of the Baltic region (Lithuania, Latvia, and Estonia) and Luxembourg populations also revealed statistically significant genetic differentiation (*Φ_CT_* = 0.247), but to a smaller extent. Genetic differentiation of similar value was found among the populations of the Baltic region (*Φ_SC_* = 0.183), divided into separate river basins within populations (*Φ_ST_* = 0.385). In all sectors grouped by distances, the genetic diversity within populations was highest.

**Figure 3:**
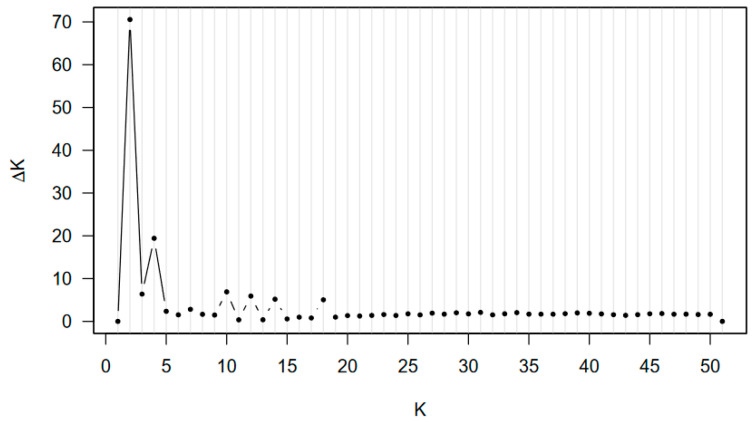
Number of Bayesian clusters of Eurasian populations of *Phalaris arundinacea* (largest ΔK = 2, second largest ΔK = 4, for selected K values from 1 to 51).

**Figure 4:**
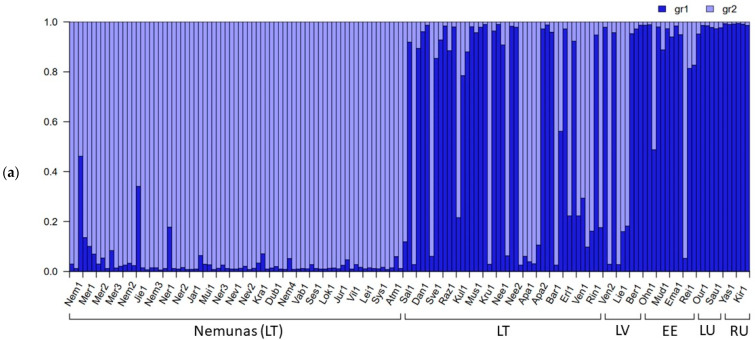

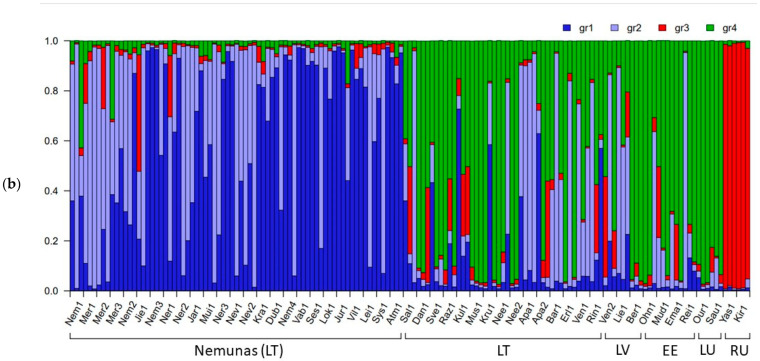
Bayesian (GBS-SNP) STRUCTURE of Lithuanian, other Baltic countries (LV, EE), Luxembourg (LU) and the Russian Far East (RU) *Phalaris arundinacea* genotypes and populations by clusters: (**a**) case of ΔK = 2 clusters, (**b**) case of ΔK = 4 clusters (columns indicate populations and columns separated by black lines indicate individuals; dark blue and light blue columns denote two (**a**) and red, green, dark blue, and light blue denote four (**b**) clusters).

Bayesian analysis showed that individuals of the 51 Eurasian populations of *P. arundinacea* consist of a mixture of primarily two (according to the largest ΔK) or four (a ΔK shoulder; Figure 3) clusters. In the case of ΔK = 2 clusters, the light blue gene cluster formed a similar part as the dark blue gene cluster among the European populations, other than the Nemunas basin (Figure 4a). Populations of the Russian Far East were distinguished by the presence of dark blue gene cluster (Figure 4a). In the case of ΔK = 4 clusters, the prevailing dark blue gene cluster separated the populations of the Nemunas basin, the red for the populations of Russian Far East, and the green for all remaining populations (Figure 4b).

### 2.3. Genetic Diversity of P. arundinacea var. picta Grown in Lithuania

**Figure 5:**
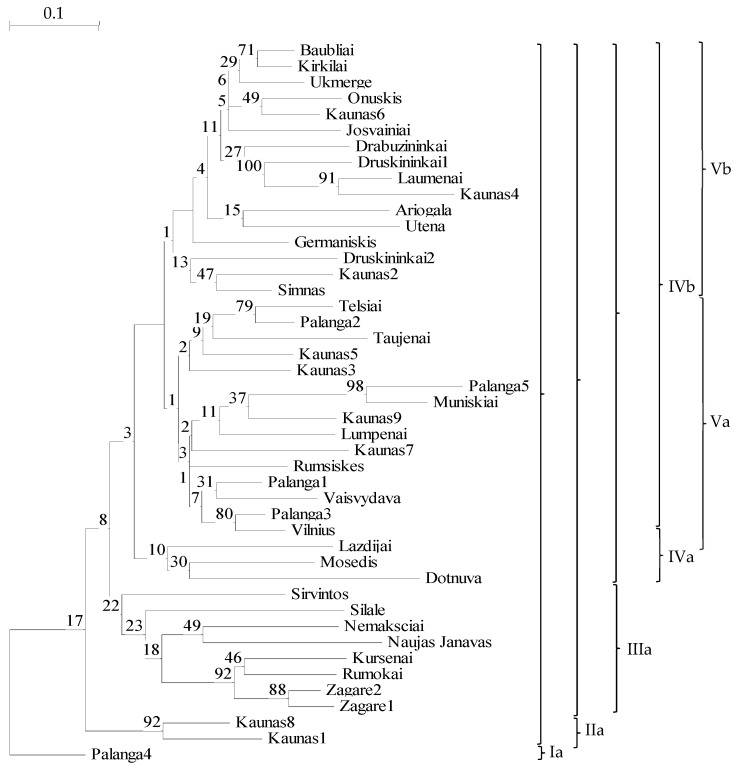
Dendrogram of genetic relationships based on 14 microsatellite markers among 45 Lithuanian ornamental form individuals of *Phalaris arundinacea* var. *picta* using the UPGMA method and Nei and Li’s [70] genetic distances with bootstrap support values (1000 replications).

Forty-five individuals of the ornamental form of *P. arundinacea* var. *picta* grown in Lithuania were analyzed by 14 microsatellite markers. The total number of alleles was n = 95. In the UPGMA dendrogram, the most distinct individuals of ornamental genotypes of *P. arundinacea* var. *picta* plants in Palanga4 (separated by the 1st order clade; Figure 5Ia), followed those in the Kaunas1 and Kaunas8 sites (separated by the 2nd order clade; Figure 5IIa).

**Figure 6:**
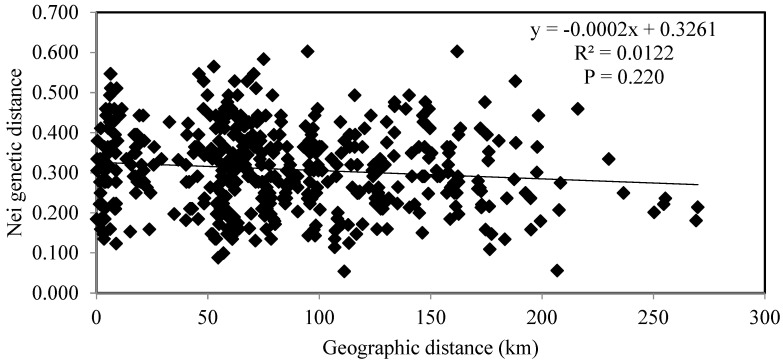
Relationship among geographical (km) and Nei’s genetic distances [70] for 14 microsatellite loci for ornamental accessions of Lithuanian *Phalaris arundinacea* var. *picta*.

There was no statistically significant relationship among the geographic distances (km) and Nei’s genetic distances (R^2^ = 0.0122) of 45 ornamental *P. arundinacea* var. *picta* genotypes growing in various parts of Lithuania (Figure 6). Linear regression formed a nearly level line, y = −0.0002x + 0.3261.

**Figure 7:**
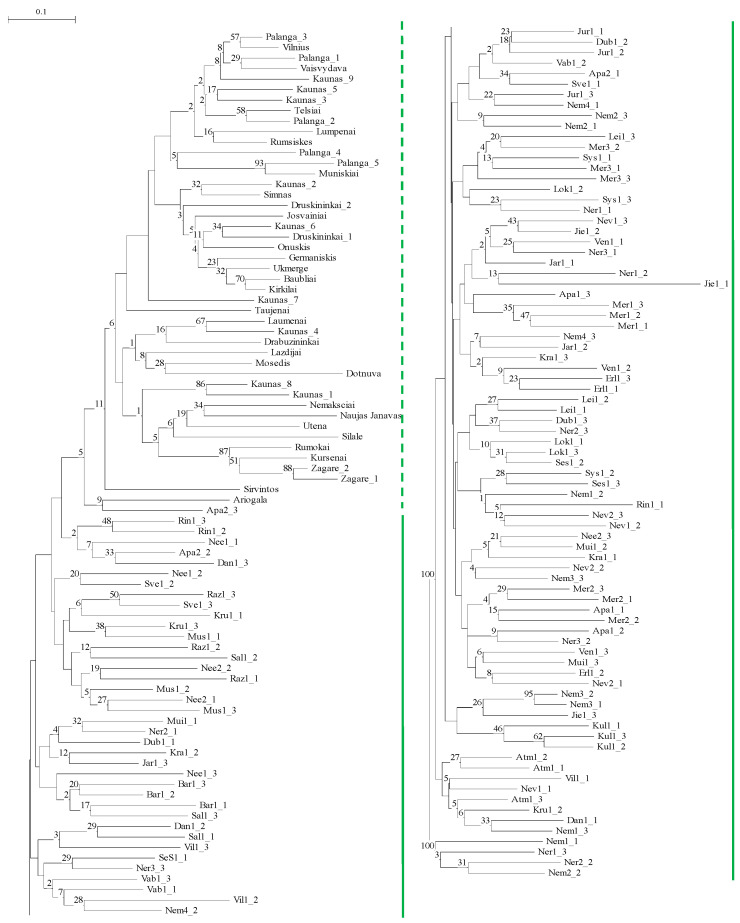
UPGMA dendrogram of genetic relatedness of genotypes of Lithuanian *Phalaris arundinacea* var. *picta* grown for ornamental purposes and naturally growing *P. arundinacea* riparian plants, constructed using Nei and Li’s [70] genetic distances according to 14 microsatellite markers (*cf*. Tables S3 and S4 for genotype descriptions). Solid green line indicates naturally growing riparian individuals; dotted line indicates cultivated ornamental genotypes). Note: due to the length of the figure, the base of the left-hand figure portion continues on the top of the right-hand figure portion.

Comparison of 45 genotypes of ornamental *P. arundinacea* var. *picta*, grown in Lithuania, and 120 genotypes of 40 riparian *P. arundinacea* populations of Lithuania revealed that genotypes of the ornamental forms and populations formed two separate clades in the UPGMA dendrogram (Figure 7).

Genetic differentiation between the groups of cultivated individuals and individuals of populations was very high (31%), although lower than within groups of natural and cultivated individuals (69%; Table 6).

**Figure 8:**
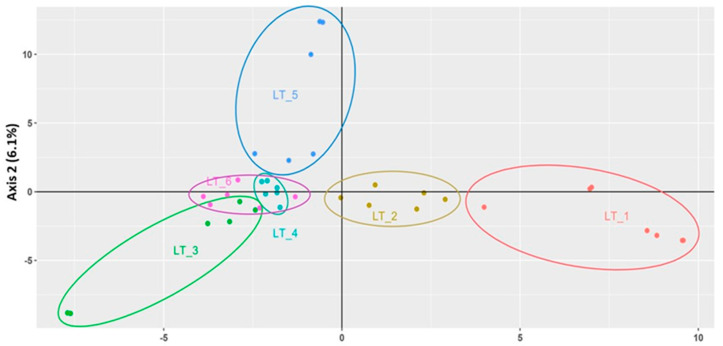
Genetic diversity plot of single nucleotide polymorphisms (SNPs) using principal component analysis, PCA (a type of principal coordinate analyses, PCoA, that uses Euclidean distance), for the first two coordinates (PCA 1, PCA 2) of clusters LT1 to LT6 populations (see text), based on single nucleotide polymorphisms (SNPs) of Lithuanian wild, riparian *Phalaris arundinacea* and cultivated *P. a.* var. *picta*. In the figure LT sites (LT 1-6) correspond to the sites of the Table S3, LT1-Ber, LT2-Mer4, LT5-Ner2, LT3-Nem1, LT4-Nem3 and LT6-Atm1.

Only 80% of the samples passed the filtration steps with high enough quality/quantity of DNA to produce enough GBS-SNPs. Thus, we have SNP analyses of 37/45 samples from the six river Lithuanian basin populations. The first two coordinates (PCA1, PCA2) of the principal component analysis (PCA) of single nucleotide polymorphisms (SNPs) accounted for a total of 12.8% of the genetic variation (Figure 8). There were ΔK = 3 clusters from the STRUCTURE analyses, grouping LT1 and LT2 groups together, along with LT5 as a separate group whereas LT3–LT6 populations were within the third, larger group (Figure 8). River basin populations LT1 (Ber) and LT2 (Mer4) [63] are genetically distinct from the remaining populations but are both grouped together.

**Figure 9:**
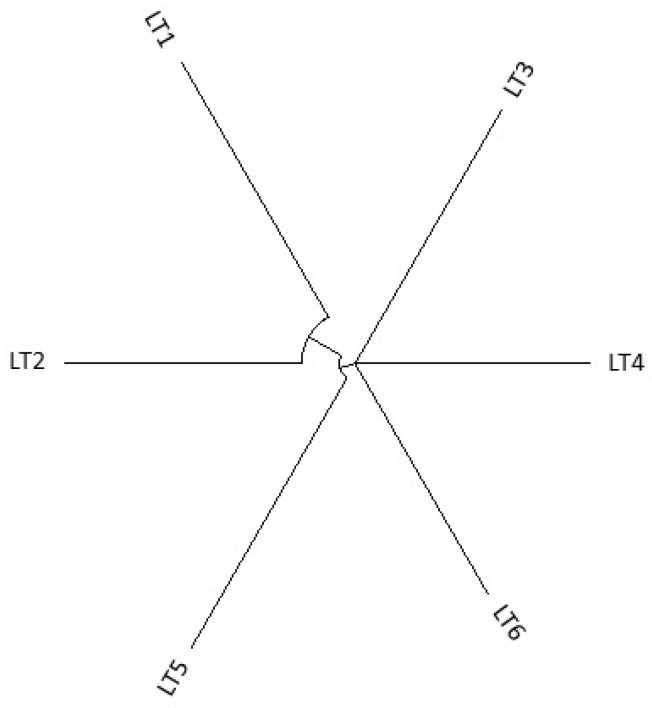
A UPGMA fan of *Phalaris arundinacea* genetic relatedness for 37 wild, extant genotypes (LT1 to LT6 populations; see text) growing in Nemunas river basin of Lithuania, constructed using Nei and Li’s genetic distance [70] from single nucleotide polymorphism (SNP) data. In the figure LT sites (LT 1–6) correspond to the sites of the Table S3, Figure 10: LT1—Ber, LT2—Mer4, LT5—Ner2, LT3—Nem1, LT4—Nem3 and LT6—Atm1.

The LT5 population (Ner2) is genetically distinct as well, as a separate group, whereas population LT3 (Nem1) overlaps slightly (is more genetically similar) with populations LT4 (Nem3) and LT6 (Atm1), both of which overlap (share SNPs in common with each other as well) (Figure 8). The river basin UPGMA dendrogram of SNPs fanned out in the same configuration as found with the ΔK = 3 clusters from the STRUCTURE analysis (Figure 9). The two most diverse genotypes in the SSR analysis (Table 2) were in two distinct but closely related clusters: highly polymorphic Jie1 (LT2) versus lowest polymorphic Lok1 (LT5) (Figure 9).

## 3. Discussion

### 3.1. Genetic Diversity of Populations

Rivers and riparian ecosystems play important ecological and economical roles in the Baltic States. Similar to previous studies on Lithuanian macrophytes [67], our sampling procedures have shown that *P. arundinacea* is widespread in all river basins of Lithuania [65,69]. Before our investigations, there were no genetic studies of *P. arundinacea* populations in the Baltic States. In parallel to our studies of populations of *P. arundinacea*, the genetic diversity parameters of some other common aquatic plant species naturally growing in Lithuania were assessed by molecular methods: *Nuphar lutea* [71], *Lythrum salicaria* [72], or *Phragmites australis* [73].

Molecular research of *P. arundinacea* is mainly focused on the invasion process of this species in N. America [12,29,33,52,57,58]. In Europe, few investigations have been devoted to examining large numbers of populations of the separate countries [17,53]. For populations of this species, a variety of molecular diversity indices have been applied: intrapopulation indicators such as percentage of polymorphic loci (PLP) [12], polymorphism information content (PIC) [55], heterozygosity [12,52] and interpopulation indicators such as phylogenetic trees [12,17,52,54], Mantel test [56,58], analysis of simple and hierarchic molecular variance [12,17,29,52,53,54,55,56,60], analysis of principal coordinates [29,52,53,55,56,57,60], as well as Bayesian structure analysis of populations [12,29,53,54,55,56,57,58,60,62]. In total, we studied 153 individuals of *Phalaris arundinacea*, while in the above-mentioned assessments of this species, the number of genotypes ranged from 30 to 900. In terms of the number of genotypes per population, studies of *P. arundinacea* ranged from 1 [60] up to 30 individuals [62]. Our study is distinguished by a very small area of examination: 120 genotypes out of 153 were sampled in Lithuania (geographic area of 65,300 km^2^).

Among microsatellite markers used in our investigation, 11 out of 15 were developed for *Phalaris canariensis* [74] and 4 markers were developed for *Zea mays* [75], while 12 out of 15 were applied for analysis of the Columbia and Missouri River Basin populations of *P. arundinacea* [60]. After our studies commenced, eight new microsatellite markers were created for *P. arundinacea* [59] and applied in the other regional investigations [29]. Future research incorporating these new markers may provide additional insights into genetic variability of *P. arundinacea*.

In this study, the total number of alleles of populations per primer ranged from 3 to 11 (in total, 95 alleles), with an average of 6.7 alleles per primer. These numbers of alleles were similar to microsatellite data of *Phalaris canariensis* (2–9 alleles, in total, 137 alleles) [75] and different from numbers of alleles obtained by Jakubowski et al. [58] in their investigation of *P. arundinacea*. The lower number of alleles in our study could be explained by the “bottle neck” effect, which might be due to the smaller number of plants collected, and/or a small geographic study area (since the main analyses were concentrated in Lithuania).

In our study, all microsatellite loci were polymorphic, as in our former investigation of the Merkys river basin [63] or in other countries [56]. In the Eurasian *P. arundinacea*, the percentage of polymorphic microsatellite loci ranged between 18.1 and 40.4% (average = 29.9%) (Table 2). The analysis of some other world regions (European and N. American populations) revealed a wider range (48–100%) of polymorphism with the microsatellite loci [56]. The greatest contrast in our study of population polymorphisms by country was between Luxembourg (33.0%) and Russian Far East (25.0%) (Table 2).

In comparable investigations of other invasive wetland species, the percentage of polymorphic AFLP loci for Lithuanian populations of *Lythrum salicaria* averaged 57.2% [72] and for Lithuanian populations of *Echinocystis lobata* was, on average, 52%, and very similarly at the regional scale in Romanian, Baltic State, and Central Russian populations averaging 51% [76]. A wider range of ISSR loci polymorphism was documented for three *Impatiens* species (13.3–67.8%) sampled in the Czech Republic and Lithuania [77].

### 3.2. Interpopulation Variability

In the present study, statistically significant relations (Mantel tests) were found among geographic and genetic distances up to 8416 km of Eurasian populations (Figure 1), similar to Jakubowski et al.’s [56] assessments of Eurasian populations within a distance up to 1000 km and European populations within distances to 250 km [58]. Mantel tests revealed a significant correlation among genetic and geographic distances of Lithuanian populations of naturally growing *Lythrum salicaria* [73] or invasive *Echinocystis lobata* [77], both supporting the theory that this exotic species is currently naturally spreading along the riverbanks.

The genetic diversity of Lithuanian populations within our study was higher within (82%) rather than among (17–18%) populations (Table 4), regardless of land cover types (2%), differing river status (1%), nitrogen concentrations (2%), river size (0%), and riverbed origins (0%). Different studies of *P. arundinacea* revealed that the intrapopulation diversity ranging within interval 62–85% [12,52,53,55,56]. High intrapopulation diversity might be explained by the high gene flow, characteristic for wind pollinated (anemophilous) species such as *Phalaris* [52]. In our Lithuanian investigation, the extent of the diversity within populations (62%) of *P. arundinacea* was intermediate compared to populations of the other Lithuanian macrophytes for microsatellite loci: diversity within populations of *Nuphar lutea* was 80% [69] and within populations of *Phragmites australis* was 46% [74]. In our Eurasian investigation, the extent of the intrapopulation diversity within *Phalaris arundinacea* populations (62%) was similar to Lithuanian populations.

Analyses of molecular variance (AMOVAs) showed Lithuanian populations of *P. arundinacea* to be significantly differentiated in respect to river basins with molecular diversity ranging from 6 to 11% of the total genetic variability (Table 3). This supports the data on Lithuanian populations of other species: *Nuphar lutea* (7% at SSR loci) [69], *Echinocystis lobata* (6–9% at AFLP loci) [76], *Lythrum salicaria* (5% at AFLP loci) [73]. Statistically significant low extent of differentiation (2% of the total genetic variability) among Lithuanian population groups of *Phalaris arundinacea*, with respect to different land cover and use class [78,79], river status (1% of the total genetic variability) [80,81], and former (1991–1996) nitrogen pollution by agriculture [82] was obtained (Table S5). Different land cover and use class were also significant at a similar extent (1% of the total genetic variability) for genetic differentiation of *Lythrum salicaria* population in Lithuania [73]. Our present study shows that land cover and use type, river status, and former nitrogen pollution in Lithuania have affected the genetic structure of populations. It may explain the above-mentioned fact that in our study, the polymorphism of populations of *P. arundinacea* was lower (29.9% on average) compared to the data (49.3% on average) of the former implemented investigation of this species in Merkys basin rivers and rivers located in protected area [63], which experienced much lower degrees of disturbance.

**Figure 10:**
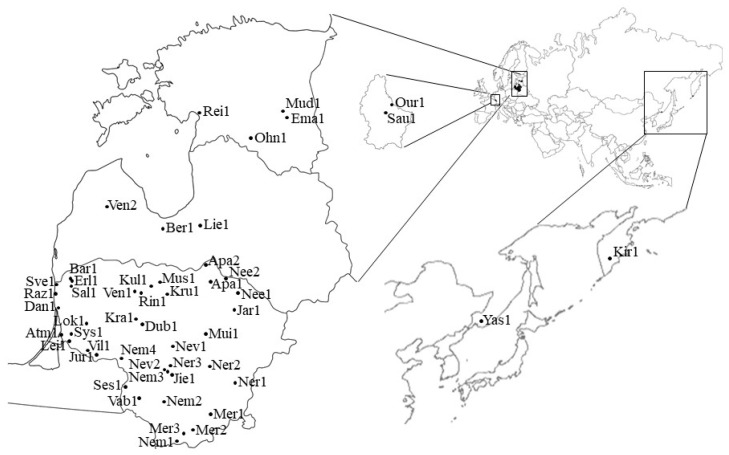
Geographical position of populations of *Phalaris arundinacea* from Lithuania (five river basins), other Baltic countries, Luxembourg, and the Russian Far East. Map source (https://vemaps.com/, accessed on 1 August 2023, https://www.freeworldmaps.net/, accessed on 1 August 2023).

For *P. arundinacea,* no significant genetic differentiation was registered among groups of populations located near distinct river sizes [80] or riverbed origins [4] (Table 4). Examination of leaf nitrogen concentrations among populations of riparian species in Lithuania also did not show differences among *P. arundinacea* populations with respect to river size. Thus, this parameter was important for only one species (*Echinocystis lobata*) out of the seven analyzed [71]. Riverbed straightening was mainly carried out for smaller rivers and rivulets in Lithuania [4]. Our present assessment of *Phalaris arundinacea* in most cases encompassed populations beside larger rivers (Figure 10, Table S3).

The number of polymorphic SSR loci was larger for populations of *P. arundinacea* on the riversides of the intact fragments of the Merkys river than for those in the regulated fragments of the riverbed [63]. Riverbed straightening has significant effects on differentiation of *Lythrum salicaria* populations among which there were more sites beside smaller rivers [73]. Statistically significant differentiation (at microsatellite loci) was not found between groups of Lithuanian population of *Phragmites australis* in relation to riverbed origin [74].

In parallel to our assessment of *P. arundinacea* relationships between genetic differentiation and different environmental variables such as temperature, humidity, nitrogen content, and riverbed origin, were documented in the USA [60]. Genetic differentiation was significant (25% of the total genetic variability, Table 5) among the populations of Romania, the Baltic States, and the Russian Far East. A similar situation was observed for *Echinocystis lobata* where differentiation was significant (12.5% of the total genetic variability at AFLP loci) among the populations of Romania, the Baltic States, and Central Russia [77]. Eurasian data for differentiation of *Phalaris arundinacea* was supported in the PCA, where populations of the Russian Far East and those of the Nemunas river basins were clearly separated from the other investigated populations (Figure 2).

In our study, the Eurasian populations consisted of two Bayesian gene clusters (Figure 3 and Figure 4). In global studies of populations, cultivars, and herbarium specimens of *P. arundinacea*, two gene pools were most often documented between the European and North American continents [12,29,54,57,60]. In other investigations within continents, three [29,57,58], four [62], six [53,57] or eight [56] gene clusters were documented. Lithuanian populations of *Lythrum salicaria* are admixtures of two gene pools of [73], and larger numbers of genetic clusters have been characteristic for populations of invasive species: three gene clusters for *Echinocystis lobata* populations [77] and *Impatiens glandulifera* [83] and multiple gene clusters for *Impatiens parviflora* for AFLP loci [73].

Similarities in the genetic makeup of the Eurasian populations of *P. arundinacea,* studied herein, with those of extensively researched N. American populations is useful for patterning future genomic and evolutionary studies of a native, yet potentially invasive, plant species. While intrapopulation genetic variation remained high for populations in both continents (82% for Eurasia, 62–85% in N. America [12,52,53,55,56]), the significant correlation of geographic and genetic distances over the 8416 km area indicates distinct origin(s) or evolved genetic makeup of the species on the local (e.g., two gene pools within Lithuania) and continental scales. This, along with the Mantel tests’ support of the theory of spread in other invasive species [73,77], evoke questions on whether differences in spread as a potentially native, invasive species are occurring. If they existed, they could vary widely among countries over the extensive geographic distances. Further research into the extent of *P. arundinacea* spread as a native yet potentially invasive Eurasian species and whether control measures are warranted in managed areas would yield intriguing comparisons with the N. American continent evoking all of the dilemmas now facing those areas [66,67].

### 3.3. Genetic Features of Ornamental GenotypesAccessions of Lithuanian P. arundinacea

Mantel tests did not show correlations for Lithuanian ornamental accessions of *Phalaris arundinacea* (Figure 6). None of the five ornamental genotypes from different private gardens in Palanga, nor nine other ornamental genotypes from different parts of Kaunas, grouped into the same clade of the phylogenetic tree (Figure 5). This may serve as evidence that in some towns and cities ornamental cultivar(s) of *P. arundinacea* were introduced from several distinct sources (differing genetic makeup) over time. Similar findings were reported with ornamental cultivars in the Czech Republic that differed genetically among genotypes within the population as well as within cultivars [53]. In the phylogenetic tree of all examined Lithuanian genotypes, the ornamental genotypes of *P. arundinacea* were distinct from those of wild, riparian populations (Figure 7). Again, similar data distinctions among groups of naturally growing and ornamental genotypes of *P. arundinacea* were found in the Czech Republic [53]. In the current study, the differences among ornamental, cultivated, and riparian plants were consistent with Jakubowski et al. [55] findings of differences among cultivars and natural European genotypes, although contradicted by some other analyzes [12], where no differences in the genetic diversity between cultivars and natural populations were documented. These discrepancies could be due to use of different molecular marker systems, the genetic makeup of the test populations, or a smaller cultivar set used by Nelson et al. [12]. In our study, ornamental genotypes were more similar to each other than those from riparian populations, presumably due to breeding and selection effects in the creation of ornamental cultivars, differing genetic backgrounds of the cultivars in comparison with wild Lithuanian populations and maintaining clonal integrity in ornamental genotype propagules.

### 3.4. River Basin Population Genetic Variation (SSRs, SNPs)

In the 1950s–1960s, many smaller rivers in Lithuania were regulated [63]. The Merkys river basin, the largest protected area of Lithuania, underwent severe anthropogenic modifications [53]. Thus, it is not surprising that the mean number of polymorphic SSR loci was lower for populations from regulated parts of the river basin compared with natural ones. Two selected populations from this basin were the Ber river fragment (regulated) and Mer4 (natural) [53]. Regulated Ber appeared to be the most distinct population in comparison to the Nemunas basin populations. The SNP data found three distinct genetic groups which consisted of river basin populations regulated LT1 (Ber), natural river fragment population LT2 (Mer4), the LT5 (Ner2) population whilst populations LT3 (Nem1), LT4 (Nem3) and LT6 (Atm1) were genetically similar (Figure 8 and Figure 9).

This research is the first regional assessment of the genetic diversity of *P. arundinacea* populations depending on environmental factors. Prior to our research, there were no publications on the impact of different classes of land cover or riverbed origin on the genetic diversity of *P. arundinacea* populations, although many studies were conducted with populations along multiple rivers in both Europe and North America [29,53]. This knowledge on the genetic diversity of *P. arundinacea* in N. America and Western and Central Europe is supplemented by our study with data from the Baltic States. For the first time, the ornamental cultivars and genotypes of *P. arundinacea* cultivated in the private gardens of Lithuanian cities and settlements were evaluated by molecular markers. The work is new in a versatile study of *P. arundinacea*, whereby different genetic, physiological, and ecological characteristics of the same populations are linked.

In our study, the genetically diverse DNA bank of Lithuanian populations of *P. arundinacea* that has been collected could be used in the future selection process to develop new productive cultivars that might be used for fodder or raw material for biofuel production. Despite the small size of the country of Lithuania, it would be expedient to use the seeds originating from local populations for the restoration of degraded lands. Our data complement the global knowledge of the genetic structure of the *P. arundinacea* within its European native range to enhance future studies of the mechanisms of species invasion.

## 4. Materials and Methods

### 4.1. Sites and Sample Collection

Research was performed in five Lithuanian river basins (Nemunas, Coastal rivers, Lielupe, Venta, Bartuva). Collection sites were located within 54°01′12.5″–56°23′51.5″ (N) and 21°03′56.4″–25°19′16.1″ (E) (Figure 10). Specific locations of populations collected within the river basins are delineated in Table S3.

Additionally, seven populations from the other Baltic countries (Latvia and Estonia), two from Luxembourg, and two from the Russian Far East were included (Figure 10). A total of 51 populations were sampled, including 40 populations from Lithuania and 11 populations from other countries (Table S3). The distance between the Lithuanian and Luxembourg populations varied between 1179 and 1441 km; the distance between the two Luxembourg populations (Our1 and Sau1) was 8 km; the distance between the Lithuanian and Russian Far Eastern populations (Yas1, Kir1), was between 7002 and 7277 km; the distance between the two Russian Far Eastern populations (Yas1 and Kir1) was 2274 km; and the distance between Luxembourg and Russian Far Eastern populations varied between 8134 and 8416 km (Table 4). From each population, three genotypes (individuals) were collected according Jakubowski et al. [12,84] resulting in a total of 153 genotypes, all of which were growing > 20 m distance apart to minimize sampling the same clones [85]: 120 from Lithuania and 33 from the other countries. Plant populations were named after river names and their abbreviations with numbers denoting each population (Table S3).

**Figure 11:**
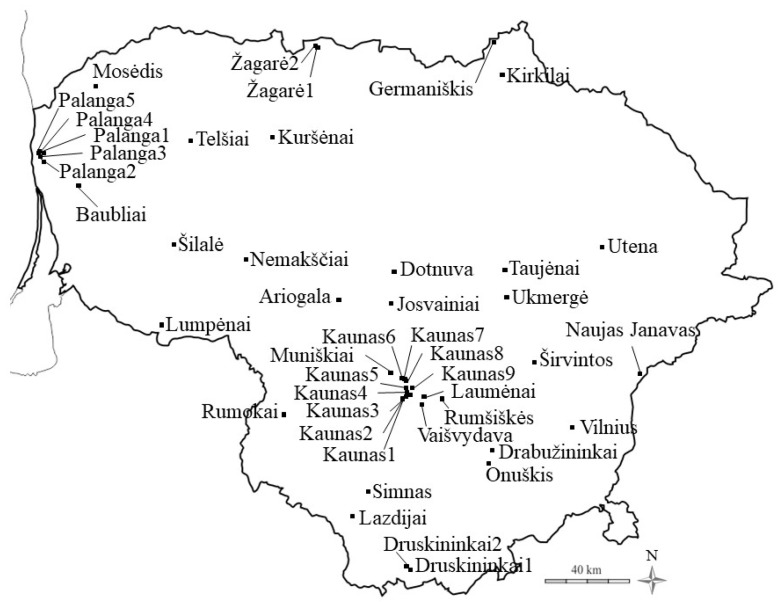
Collection sites of ornamental cultivars (genotypes) of *Phalaris arundinacea*, *P. a.* var. *picta* in Lithuania. Map source (https://vemaps.com/, accessed on 15 May 2023, https://www.freeworldmaps.net/, accessed on 15 May 2023).

For genetic analysis of ornamental genotypes, *P. arundinacea* cultivars were collected from 45 private homesteads of settlements and cities of Lithuania (Figure 11).

For GBS-SNP analysis using DArTSeq (https://www.diversityarrays.com/services/dartseq/, 15 May 2023), six populations of the Nemunas basin were selected: the upper part of the Nemunas River (Lithuanian part, LT3), the middle part of the Nemunas River (LT4), and the Atmata branch of the Nemunas Delta (LT6), as well as the population from the largest tributary of the Nemunas–Neris (LT5), and two populations from the rivers of the Nemunas tributary and the Merkys river basin (LT1 and LT2), previously studied by microsatellites [63]. Among the GBS-SNPs selected for analysis, all populations were near natural riverbeds, except for the LT1 population (regulated riverbed; Table S3).

Ornamental genotypes of *P. arundinacea* were coded after the site names and with sequential, numerical coding (Table S4). One plant (genotype) was taken from each homestead, as usually only one dense stand was grown in each landscape for a total of 45 samples collected. Plant population names, with numbers denoting each population, are delineated along with their geographic locations of latitude and longitude coordinates as well as altitudes (Table S4). The ancestral origin of each ornamental type was unknown regarding whether it was from an ornamental breeding program or a selection from the wild. Healthy, recently expanded leaves were collected, as described in Anderson et al. [63]. This maximized DNA quantity during extraction.

### 4.2. Environmental Variables

Disclosing possible effects of the river and its environment on nutritional state of the plant, Lithuanian populations of *P. arundinacea* were grouped in several ways: (1) three groups, according to the type of land cover and use class: artificial surfaces (ART), agricultural areas (AGR), forest, and semi-natural areas (FOR) [79]; (2) five groups, according to the present river state: high (H), good (G), moderate (MO), poor (P), bad (B) [78,79]; (3) three groups: populations of North-West (NW), Central (C) and South-East (SE) of Lithuania, based on N concentrations in the rivers neighboring agricultural areas during 1991–1996 [82]; (4) four groups, based on the river size: small (S, <100 km^2^), medium sized (M, 100–1000 km^2^), large (L, 1000–10,000 km^2^), and extra-large (XL, >10,000 km^2^) rivers (EU, 2000); and (5) two groups, based on riverbed origin: natural and regulated [12] (Table S5).

### 4.3. Molecular Analysis

Total genomic DNA of *P. arundinacea* was isolated using the modified CTAB method [73,86,87], with details described by Štorchová et al. [88] and some further updates [63,89]. Agarose gel (1%) electrophoresis and UV spectrophotometry (BioSpec-Nano, Shimadzu, Carlsbad, CA, USA) were used to assess DNA quality and quantity.

For molecular analysis of *P. arundinacea*, simple sequence repeat (SSR) markers or microsatellites were selected following Lawrence et al. [76], Li et al. [75], and Jakubowski et al. [56]. For the SSRs assessment, 14 primer pairs were selected for the population analyses of *P. arundinacea,* as in previous research [63]. For microsatellite analysis, amplification products were mixed with Hi-Di Formamide and GeneScanTM 500 LIZ size standard (Applied Biosystems, Warrington, UK) and denaturated at 95 °C for 3 min. Capillary gel electrophoresis was performed on an ABI Prism 3130xl Genetic Sequencer (Genetic analyzer 3130; Applied Biosystems, Darmstadt, Germany). Data were analyzed by GeneMapper v. 4.0 (Applied Biosystems, Darmstadt, Germany).

For genotype by sequencing (GBS) to generate low density single nucleotide polymorphisms (SNPs), leaf samples were dried in Lithuania and shipped to the University of Minnesota for processing, i.e., samples were ground in a Geno/Grinder^®^ Tissue Homogenizer, SPEX SamplePrep (Avantor, VWR), and ground leaf powder samples (10–20 mg/genotype) were sent to DArTSeq for DNA extraction, GBS, and SNP generation.

### 4.4. Statistical Analysis

DNA fragments, obtained for each SSR loci, were scored for presence or absence [76]. Due to possible differences in ploidy and allele abundance, the data of capillary electrophoresis were evaluated as dominant (not as codominant) following Jakubowski et al. [55] and Ketternring et al.’s [60] investigations of *P. arundinacea*.

Analysis of hierarchical molecular variance (AMOVA) among populations and within populations, along with the Mantel test for relations between genetic and geographical distances of populations and principal coordinate analysis were performed using GenAlEx v. 6.5 [90]; polymorphism of DNA, Nei’s [91] genetic diversity, Shannon’s information index [92], and Nei’s [93] genetic distances (by UPGMA) were calculated by POPGENE 1.32 [70]; Nei and Li [94]-based genetic distances for individuals by UPGMA were analyzed by TreeCon 1.3 [95]; Bayesian clustering analysis was conducted using STRUCTURE 3.2.4 [96,97]; comparison of nitrogen concentrations was completed by R 3.4.4 [98]; comparison of population groups depending on environment was performed by R package PMCMR [99] and CompareGroups [100]; and multiple correspondence analysis between groups of populations of different plant species, formed depending on certain factors, was conducted using FactoMineR [101] and FactoExtra [101].

To analyze SNP data from DArTseqLD™ genotyping for river basin populations LT1–LT6, the package dartR in R Studio was used [102]. Running gl.report.callrate, the call rates were analyzed for histograms, loci and genotypes, and selecting a call rate which maintained the highest number of genotypes while maintaining high-quality loci. SNP data were analyzed with R Studio (Version 4.2.3) was used to determine genetic diversity (principal coordinate analysis, PCA), genetic STRUCTURE (Version 1.2.5033) and STRUCTURE Harvester, and GenAlEx 6.5 [90] using Microsoft Excel.

## 5. Conclusions

This analysis of *P. arundinacea* populations across European and Eurasian countries provides evidence that the species is widespread across the continent in its native riparian habitat. Genetic variation, based on 14 tested *P. canariensis* microsatellites, demonstrated the highest variability was within (82%) populations, rather than among (18%), which matches previous extensive studies in N. American populations. This variation occurred regardless of land cover types (2%), differing river status (1%), nitrogen concentrations (2%), river size (0%), or riverbed origin (0%). Genetic differentiation was significant (25% of the total genetic variability) among the populations of Romania, the Baltic States, and the Russian Far East. In Eurasia, intrapopulation diversity within *P. arundinacea* populations (62%) was similar to Lithuanian populations. Such high intrapopulation diversity may be due to high gene exchange, characteristic for wind pollinated species such as *Phalaris.*

Allelic numbers among populations totaled 95 alleles, averaging 6.7 alleles/primer, and were slightly lower than previous findings. We theorize this reduction to be due to “bottle neck” effects.

All microsatellite loci were polymorphic, e.g., the Eurasian percentage of polymorphic microsatellite loci averaged 29.9%, significantly less than other European and N. American populations. The greatest difference polymorphisms per country was between the Russian Far East (25.0%) and Luxembourg (33.0%).

Statistically significant correlations among genetic and geographic distances up to 8416 km of Eurasian populations were similar to previous findings for other populations, supporting the theory that this species, while native to Europe and Eurasia, continues natural spreading along riverbanks as a potentially invasive–yet native–species. Implications of this research will be widespread in applicability to future genomic-based evolutionary biology studies.

Lithuanian populations are differentiated among river basins (6–11% of total genetic variability). Land cover and use type, river status, and former nitrogen pollution in Lithuania affected the genetic structure of populations.

Ornamental genotypes (cultivars) were more similar to each other than those from riparian populations, potentially due to breeding and selection effects. Maintaining clonal integrity in ornamental cultivars would maintain this genetic differentiation from wild native, riparian populations.

## Figures and Tables

**Table 1:** SSR markers used for analysis of populations of *Phalaris arundinacea*, the number of alleles generated by each microsatellite primer pair, and allele size range.

Primer	Number of Alleles	Range of Allele Size (bp)
csm045	9	90–106
csm049	3	144–160
csm071	5	126–160
csm074	5	115–151
csm075	11	133–161
csm090	4	97–105
csm101	5	113–149
csm104	4	104–110
csm106	13	131–163
csm111	5	107–138
csm122	5	121–149
phi071	10	126–154
umc2185	10	104–155
umc2779	6	121–147
Total	95	

**Table 2:** Microsatellite loci-based genetic diversity of *Phalaris arundinacea* populations of the river basins of Lithuania, other Baltic States, Luxembourg, and Russian Far East: % P (percentage of polymorphic DNA loci), *I*—Shannon’s information index, and *h*—index of Nei’s gene diversity.

Populations	% P	*I*		*h*	
		Mean ± SE		Mean ± SE	
Europe	30.0	0.166	±0.027	0.112	±0.018
Russian Far East	25.0	0.139	±0.025	0.093	±0.017
Baltic countries	30.1	0.169	±0.027	0.114	±0.019
Lithuanian	29.5	0.166	±0.027	0.112	±0.018
Nemunas basin (LT)	29.5	0.166	±0.027	0.122	±0.019
Seaside river basin (LT)	32.2	0.176	±0.027	0.117	±0.018
Lielupė basin (LT-LV)	28.6	0.163	±0.027	0.110	±0.019
Venta basin (LV)	33.0	0.199	±0.029	0.135	±0.020
Bartuva (LT)	22.9	0.130	±0.025	0.088	±0.018
Peipus (EE)	33.5	0.182	±0.027	0.121	±0.018
Rein (LU)	33.0	0.182	±0.028	0.122	±0.019
Average	29.9	0.168	±0.004	0.113	±0.003

**Table 3:** Microsatellite loci-based hierarchical analysis of molecular variance (AMOVA): among populations from the Nemunas and other Lithuanian river basins, among populations within basins, and within populations of *Phalaris arundinacea*.

Source	df	SS	Est. Var.	%	*Φ*
1.					
Among groups of populations of Nemunas and Lielupė basins	1	49	0.97	8	*Φ*_CT_ = 0.083 ***
Among populations within groups	31	429	1.56	13	*Φ*_SC_ = 0.145 ***
Within populations	66	605	9.16	78	*Φ*_ST_ = 0.216 ***
Total	98	1082	11.69	100	
2.					
Among groups of populations of Nemunas and Seaside basins	1	35	1.03	9	*Φ*_CT_ = 0.088 ***
Among populations withing groups	27	360	1.33	11	*Φ*_SC_ = 0.125 ***
Within populations	58	542	9.35	80	*Φ*_ST_ = 0.201 ***
Total	86	937	11.70	100	
3.					
Among groups of populations of Nemunas and Venta basin	1	25	0.70	6	*Φ*_CT_ = 0.061 ***
Among populations within groups	26	348	1.32	12	*Φ*_SC_ = 0.123 ***
Within populations	56	527	9.42	82	*Φ*_ST_ = 0.176 ***
Total	83	900	11.44	100	
4.					
Among groups of populations of Nemunas and Bartuva basins	1	28	1.35	11	*Φ*_CT_ = 0.113 ***
Among populations within groups	25	336	1.45	12	*Φ*_SC_ = 0.138 ***
Within populations	54	490	9.07	76	*Φ*_ST_ = 0.236 ***
Total	80	854	11.87	100	
5.					
Among groups of populations of different Baltic States river basins (Nemunas, Seaside rivers, Lielupė, Bartuva, Venta, Dauguva, Vorstjarv, Peipus, Pernu)	8	185	0.79	7	*Φ*_CT_ = 0.068 ***
Among populations within groups	38	523	1.46	12	*Φ*_SC_ = 0.134 ***
Within populations	94	884	9.40	81	*Φ*_ST_ = 0.193 ***
Total	140	1592	11.65	100	

df—degrees of freedom; SS—sum of squares; Est. Var.—estimated variability; %—percentage of variation; *Φ*—pairwise population genetic distances: proportion of variance among the river basins (*Φ*_ST_), among populations (*Φ*_SC_) and within populations (*Φ*_CT_), *** *p* ≤ 0.001; Lielupė and Venta river basin populations had one population from Latvia.

**Table 4:** Microsatellite loci-based hierarchical analysis of molecular variance (AMOVA) of Lithuanian populations of *Phalaris arundinacea*: within populations, among populations, and among various groups of populations depending on habitat features.

Source	df	SS	MS	Est. Var.	%	*Φ*
1.						
Among groups of populations beside different land cover types	2	43	21.7	0.197	2	*Φ*_CT_ = 0.017 ***
Among populations	37	550	14.9	1.882	17	*Φ*_SC_ = 0.169 ***
Within populations	80	738	9.2	9.225	82	*Φ*_ST_ = 0.184 ***
Total	119	1332		11.304	100	
2.						
Among groups of populations beside different river state	3	54	17.8	0.106	1	*Φ*_CT_ = 0.009 *
Among populations	36	540	15.0	1.925	17	*Φ*_SC_ = 0.173 ***
Within populations	80	738	9.2	9.225	82	*Φ*_ST_ = 0.180 ***
Total	119	1332		11.256	100	
3.						
Among groups of populations from different areas based on N concentrations within 1991–1996	2	45	22.3	0.197	2	*Φ*_CT_ = 0.017 ***
Among populations	37	549	14.8	1.870	17	*Φ*_SC_ = 0.169 ***
Within populations	80	738	9.2	9.225	82	*Φ*_ST_ = 0.183 ***
Total	119	1332		11.192	100	
4.						
Among groups of populations beside different size rivers	3	47	15.6	0.014	0	*Φ*_CT_ = 0.001 ^NS^
Among populations	36	547	15.2	1.989	18	*Φ*_SC_ = 0.177 ***
Within populations	80	738	9.2	9.225	82	*Φ*_ST_ = 0.178 ***
Total	119	1332		11.228	100	
5.						
Among groups of populations beside different riverbed origins	1	13	12.5	0.000	0	*Φ*_CT_ = −0.006 ^NS^
Among populations	38	581	15.3	2.022	18	*Φ*_SC_ = 0.180 ***
Within populations	80	738	9.2	9.225	82	*Φ*_ST_ = 0.175 ***
Total	119	1332		11.247	100	

df—degrees of freedom; SS—sum of squares; MS—mean squares; Est. Var.—estimated variability; %—percentage of variation; *Φ*—pairwise population genetic distances: proportion of variance among the river basins (*Φ*_ST_), among populations (*Φ*_SC_) and within populations (*Φ*_CT_). 1. Land cover types: (ART)—artificial surfaces, (AGR)—agricultural areas, (FOR)—forest and semi-natural areas; 2. River state: (H)—high, (G)—good, (MO)—moderate, (P)—poor, (B)—bad; 3. Geographic area based on N concentrations: 1—North-West of Lithuania, 2—central Lithuania, 3—South-East of Lithuania; 4. River size: small (S, <100 km^2^), medium (M, 100–1000 km^2^), large (L, 1000–10,000 km^2^), and extra-large (XL, >10,000 km^2^); 5. Riverbed origin: (N)—natural, (R)—regulated. * *p* ≤ 0.05, *** *p* ≤ 0.001; NS—statistically non-significant.

**Table 5:** Microsatellite loci-based hierarchic AMOVA (analysis of molecular variance) of Eurasian populations of *Phalaris arundinacea* between the European and Asian continents, and between Europe and the Baltic region, in four groups based on distances apart (>1000 km, >7000 km, >8000 km transects) among groups of populations, among populations, and within populations.

Source	df	SS	Est. Var.	%	*Φ*
1. >8000 km transect					
Among groups of populations of Europe and Asia continents (49 + 2)	1	59	3.77	25	*Φ*_CT_ = 0.247 ***
Among populations within Europe and Asia population groups	49	769	2.10	14	*Φ*_SC_ = 0.183 ***
Within populations	102	957	9.38	62	*Φ*_ST_ = 0.385 ***
Total	152	1784	15.25	100	
2. >8000 km transect					
Among groups of populations of Luxembourg and Russian Far East (2 + 2)	1	39	3.58	23	*Φ*_CT_ = 0.230 *
Among populations within LU and Russian Far East population groups	2	36	2.92	19	*Φ*_SC_ = 0.243 *
Within populations	8	73	9.08	58	*Φ*_ST_ = 0.417 ***
Total	11	148	15.58	100	
3. >7000 km transect					
Among groups of populations of Nemunas and Russian Far East (25 + 2)	1	66	4.66	30	*Φ*_CT_ = 0.303 ***
Among populations within Nemunas and Far East population groups	25	348	1.60	10	*Φ*_SC_ = 0.149 ***
Within populations	54	493	9.12	59	*Φ*_ST_ = 0.407 ***
Total	80	906	15.38	100	
4. >1000 km transect					
Among groups of populations of Baltic region and Luxembourg (47 + 2)	1	25	0.81	7	*Φ*_CT_ = 0.066 *
Among populations within Baltic countries and LU population groups	47	719	1.95	16	*Φ*_SC_ = 0.171 ***
Within populations	98	925	9.44	77	*Φ*_ST_ = 0.226 ***
Total	146	1669	12.20	100	

df—degrees of freedom; SS—sum of squares; Est. Var.—estimated variability; %—percentage of variation; *Φ*—pairwise population genetic distances: proportion of variance among regions (*Φ*_ST_), among populations (*Φ*_SC_) and within populations (*Φ*_CT_), * *p* ≤ 0.05, *** *p* ≤ 0.001.

**Table 6:** Microsatellite loci-based molecular variance of Lithuanian ornamental individual genotypes grown and naturally growing individuals of *Phalaris arundinacea*.

Source	df	SS	Est. Var.	%	*Φ*
Among groups of grown and naturally growing individuals	1	349	348.6	31	0.306 ***
Within populations	163	1902	11.7	69	
Total	164	2251		100	

df—degrees of freedom; SS—sum of squares; Est. Var.—estimated variability; %—percentage of variation; *Φ*—pairwise population genetic distances, *** *p* ≤ 0.001.

## Data Availability

The data presented in this study are available on request from the corresponding author due to privacy.

## Supplementary Materials

The following supporting information can be downloaded at https://www.mdpi.com/article/10.3390/genes15060734/s1: Table S1. Fourteen microsatellite (SSR) loci-based indices of genetic diversity of Eurasian populations of *Phalaris arundinacea*, separated by country of origin (LT = Lithuania; EE = eastern Europe, Baltic countries; LU = Luxembourg; RU = Russian Far East), river basin, population, the number of unique alleles (N_p_), the total number of alleles (N_t_), the number of polymorphic alleles (N), percentage of polymorphic DNA loci (%P), Shannon‘s information index (*I*) and index of Nei‘s gene diversity (*h*); Table S2. Geographical (km) and genetic (Nei, 1978) distances between 51 Eurasian populations of *Phalaris arundinacea* based on 14 microsatellite markers; Table S3. Geographical locations of Eurasian *Phalaris arundinacea* populations used for genetic analysis: population code, DNA marker type, river name, river basin name, site name, country of origin, and geographical location specifics (latitude, longitude, altitude); Table S4. Site information (population numerical codes, geographic locations of latitude and longitude, and altitude) of 45 Lithuanian genotype accessions of ornamental *Phalaris arundinacea* var. *picta* or *P. arundinacea;* Table S5. Characteristics of the environment factors for Lithuanian *Phalaris arundinacea* populations used for genetical analysis: population, land cover type, river state, geographical areas based on N concentration in years 1992–1996, river size, and riverbed origin.
