# Supplementary material for: Genetic Differentiation of Reed Canarygrass (Phalaris arundinacea L.) within Eastern Europe and Eurasia
Source: Genes (Basel). 2024 Jun 3;15(6):734. doi: 10.3390/genes15060734 (PMC11202989; doi:10.3390/genes15060734)
Supplement: Supplementary file 1 [file genes-15-00734-s001.zip › genes-2998050-supplementary.pdf]

genes MDPI

**Table S1.** Fourteen microsatellite (SSR) loci-based indices of genetic diversity of Eurasian populations of *Phalaris arundinacea*, separated by country of origin (LT=Lithuania; EE=eastern Europe, Baltic countries; LU=Luxembourg; RU= Russian Far East), river basin, population, the number of unique alleles ($N_p$), the total number of alleles ($N_t$), the number of polymorphic alleles (N), percentage of polymorphic DNA loci (%P), Shannon's information index (*I*) and index of Nei's gene diversity (*h*).

| Country | River basin | Population | $N_p$ | $N_t$ | N | % P | *I* | | *h* | |
|---|---|---|---|---|---|---|---|---|---|---|
| | | | | | | | Mean | ±SE | Mean | ±SE |
| LT | Nemunas | Nem1 | 1 | 48 | 29 | 30.9 | 0.169 | ±0.027 | 0.112 | ±0.018 |
| LT | Nemunas | Mer1 | 0 | 37 | 20 | 21.3 | 0.125 | ±0.025 | 0.086 | ±0.018 |
| LT | Nemunas | Mer2 | 0 | 44 | 24 | 25.5 | 0.148 | ±0.027 | 0.101 | ±0.018 |
| LT | Nemunas | Mer3 | 1 | 51 | 31 | 33.0 | 0.190 | ±0.029 | 0.129 | ±0.020 |
| LT | Nemunas | Nem2 | 1 | 46 | 30 | 31.9 | 0.183 | ±0.028 | 0.124 | ±0.020 |
| LT | Nemunas | Jie1 | 0 | 50 | 38 | 40.4 | 0.230 | ±0.030 | 0.155 | ±0.020 |
| LT | Nemunas | Nem3 | 0 | 47 | 23 | 24.5 | 0.134 | ±0.025 | 0.089 | ±0.017 |
| LT | Nemunas | Ner1 | 0 | 50 | 34 | 36.2 | 0.209 | ±0.030 | 0.142 | ±0.020 |
| LT | Nemunas | Ner2 | 0 | 45 | 28 | 29.7 | 0.166 | ±0.027 | 0.111 | ±0.018 |
| LT | Nemunas | Jar1 | 0 | 49 | 26 | 27.7 | 0.147 | ±0.025 | 0.097 | ±0.017 |
| LT | Nemunas | Mui1 | 0 | 48 | 29 | 30.9 | 0.171 | ±0.027 | 0.114 | ±0.019 |
| LT | Nemunas | Ner3 | 0 | 43 | 26 | 27.7 | 0.160 | ±0.027 | 0.109 | ±0.019 |
| LT | Nemunas | Nev1 | 0 | 49 | 34 | 36.2 | 0.201 | ±0.028 | 0.134 | ±0.019 |
| LT | Nemunas | Nev2 | 0 | 41 | 22 | 23.4 | 0.129 | ±0.025 | 0.086 | ±0.017 |
| LT | Nemunas | Kra1 | 0 | 51 | 32 | 34.0 | 0.184 | ±0.027 | 0.122 | ±0.018 |
| LT | Nemunas | Dub1 | 0 | 47 | 29 | 31.9 | 0.178 | ±0.028 | 0.120 | ±0.019 |
| LT | Nemunas | Nem4 | 0 | 46 | 29 | 30.9 | 0.171 | ±0.027 | 0.114 | ±0.019 |
| LT | Nemunas | Vab1 | 0 | 43 | 24 | 25.5 | 0.150 | ±0.027 | 0.103 | ±0.019 |
| LT | Nemunas | Ses1 | 0 | 44 | 26 | 27.7 | 0.154 | ±0.026 | 0.103 | ±0.018 |
| LT | Nemunas | Lok1 | 0 | 40 | 17 | 18.1 | 0.104 | ±0.023 | 0.070 | ±0.016 |
| LT | Nemunas | Jur1 | 0 | 44 | 25 | 26.6 | 0.151 | ±0.027 | 0.102 | ±0.018 |
| LT | Nemunas | Vil1 | 0 | 49 | 36 | 38.3 | 0.213 | ±0.029 | 0.143 | ±0.020 |
| LT | Nemunas | Lei1 | 0 | 49 | 31 | 33.0 | 0.192 | ±0.029 | 0.131 | ±0.020 |
| LT | Nemunas | Sys1 | 0 | 46 | 30 | 31.9 | 0.189 | ±0.029 | 0.130 | ±0.020 |
| LT | Nemunas | Atm1 | 0 | 44 | 18 | 19.2 | 0.113 | ±0.025 | 0.077 | ±0.017 |
| LT | Seaside rivers | Sal1 | 0 | 46 | 29 | 30.9 | 0.164 | ±0.026 | 0.108 | ±0.018 |
| LT | Seaside rivers | Dan1 | 0 | 45 | 27 | 28.7 | 0.143 | ±0.024 | 0.092 | ±0.015 |
| LT | Seaside rivers | Sve1 | 0 | 45 | 30 | 31.9 | 0.185 | ±0.029 | 0.126 | ±0.020 |
| LT | Seaside rivers | Raz1 | 0 | 48 | 35 | 37.2 | 0.210 | ±0.029 | 0.142 | ±0.020 |
| LT | Bartuva | Bar1 | 0 | 41 | 23 | 24.5 | 0.141 | ±0.026 | 0.095 | ±0.018 |
| LT | Bartuva | Erl1 | 0 | 40 | 20 | 21.3 | 0.119 | ±0.024 | 0.080 | ±0.017 |
| LT | Lielupe | Kul1 | 0 | 42 | 20 | 21.3 | 0.132 | ±0.027 | 0.092 | ±0.019 |
| LT | Lielupe | Mus1 | 0 | 46 | 23 | 24.5 | 0.130 | ±0.024 | 0.085 | ±0.016 |
| LT | Lielupe | Kru1 | 0 | 37 | 22 | 23.4 | 0.144 | ±0.027 | 0.100 | ±0.019 |
| LT | Lielupe | Nee1 | 0 | 45 | 31 | 33.0 | 0.188 | ±0.028 | 0.127 | ±0.020 |
| LT | Lielupe | Nee2 | 0 | 47 | 28 | 29.8 | 0.166 | ±0.027 | 0.111 | ±0.018 |
| LT | Lielupe | Apa1 | 0 | 50 | 29 | 30.9 | 0.167 | ±0.026 | 0.110 | ±0.018 |
| LT | Lielupe | Apa2 | 0 | 51 | 34 | 36.2 | 0.205 | ±0.029 | 0.138 | ±0.020 |
| LT | Venta | Ven1 | 0 | 45 | 30 | 33.0 | 0.192 | ±0.029 | 0.131 | ±0.020 |
| LT | Venta | Rin1 | 0 | 49 | 33 | 35.1 | 0.202 | ±0.029 | 0.137 | ±0.020 |
| LV | Lielupe | Lie1 | 0 | 46 | 28 | 29.8 | 0.168 | ±0.027 | 0.113 | ±0.019 |

| | | | | | | | | | | |
|---|---|---|---|---|---|---|---|---|---|---|
| LV | Venta | Ven2 | 0 | 50 | 35 | 37.2 | 0.204 | ±0.028 | 0.136 | ±0.019 |
| LV | Dauguva | Kau1 | 0 | 47 | 30 | 31.9 | 0.183 | ±0.028 | 0.124 | ±0.020 |
| EE | Vortsjarv | Ohn1 | 0 | 51 | 31 | 33.0 | 0.188 | ±0.028 | 0.127 | ±0.020 |
| EE | Peipus | Mud1 | 0 | 46 | 37 | 39.4 | 0.220 | ±0.029 | 0.148 | ±0.020 |
| EE | Peipus | Ema1 | 0 | 43 | 26 | 27.7 | 0.143 | ±0.024 | 0.093 | ±0.016 |
| EE | Pernu | Rei1 | 0 | 49 | 32 | 34.0 | 0.193 | ±0.028 | 0.130 | ±0.020 |
| LU | Rein | Our1 | 0 | 47 | 29 | 30.9 | 0.171 | ±0.027 | 0.114 | ±0.019 |
| LU | Rein | Sau1 | 0 | 48 | 33 | 35.1 | 0.193 | ±0.028 | 0.129 | ±0.019 |
| RU | Yasneniya | Yas1 | 0 | 42 | 19 | 20.2 | 0.107 | ±0.022 | 0.071 | ±0.015 |
| RU | Kirpichnaya | Kir1 | 2 | 44 | 27 | 29.8 | 0.170 | ±0.028 | 0.115 | ±0.019 |
| | | Average | | | | 29.9 | 0.168 | ±0.004 | 0.113 | ±0.003 |

**Table S2.** Geographical (km) and genetic (Nei, 1978) distances between 51 Eurasian populations of *Phalaris arundinacea* based on 14 microsatellite markers.

| | Geographical distances (km) | | | | | | | | | | | | | | | | | | | | | | | | | | | | | | | | | | | | | | | | | | | | | | | | | |
|---|---|---|---|---|---|---|---|---|---|---|---|---|---|---|---|---|---|---|---|---|---|---|---|---|---|---|---|---|---|---|---|---|---|---|---|---|---|---|---|---|---|---|---|---|---|---|---|---|---|---|
| Genetic distances | Nem1 | Mer1 | Mer2 | Mer3 | Nem2 | Jie1 | Nem3 | Ner1 | Ner2 | Jar1 | Mui1 | Ner3 | Nev1 | Nev2 | Kra1 | Dub1 | Nem4 | Vab1 | Ses1 | Lok1 | Jur1 | Vil1 | Lei1 | Sys1 | Atm1 | Sal1 | Dan1 | Sve1 | Raz1 | Bar1 | Erl1 | Kul1 | Mus1 | Kru1 | Nee1 | Nee2 | Apa1 | Apa2 | Lie1 | Ven1 | Rin1 | Ven2 | Kau1 | Ohn1 | Mud1 | Ema1 | Rei1 | Our1 | Sau1 | Yas1 | Kir1 |
| Nem1 | - | 71 | 36 | 20 | 57 | 91 | 97 | 119 | 113 | 206 | 160 | 107 | 142 | 102 | 187 | 164 | 144 | 82 | 110 | 200 | 168 | 183 | 214 | 211 | 227 | 274 | 261 | 290 | 281 | 284 | 280 | 225 | 234 | 218 | 242 | 248 | 246 | 269 | 311 | 231 | 225 | 351 | 311 | 459 | 521 | 514 | 484 | 1295 | 1302 | 7242 | 7260 |
| Mer1 | 0.122 | - | 35 | 51 | 67 | 80 | 86 | 50 | 65 | 150 | 113 | 87 | 117 | 93 | 187 | 158 | 160 | 95 | 137 | 213 | 192 | 208 | 241 | 235 | 254 | 282 | 280 | 303 | 297 | 289 | 286 | 205 | 210 | 190 | 189 | 198 | 202 | 225 | 282 | 220 | 211 | 341 | 271 | 409 | 468 | 460 | 444 | 1361 | 1368 | 7171 | 7198 |
| Mer2 | 0.109 | 0.116 | - | 17 | 49 | 76 | 83 | 83 | 84 | 175 | 133 | 89 | 124 | 90 | 182 | 155 | 146 | 80 | 118 | 202 | 175 | 191 | 224 | 219 | 237 | 274 | 267 | 293 | 286 | 283 | 279 | 210 | 218 | 200 | 213 | 220 | 221 | 244 | 293 | 221 | 213 | 342 | 288 | 432 | 492 | 485 | 461 | 1328 | 1335 | 7206 | 7228 |
| Mer3 | 0.102 | 0.164 | 0.130 | - | 46 | 77 | 84 | 99 | 94 | 186 | 142 | 92 | 128 | 90 | 179 | 154 | 140 | 75 | 109 | 196 | 167 | 183 | 214 | 211 | 228 | 269 | 259 | 287 | 279 | 278 | 275 | 212 | 221 | 204 | 223 | 230 | 229 | 252 | 297 | 220 | 214 | 342 | 295 | 441 | 502 | 495 | 468 | 1312 | 1318 | 7222 | 7240 |
| Nem2 | 0.096 | 0.135 | 0.107 | 0.101 | - | 34 | 40 | 95 | 69 | 159 | 111 | 51 | 86 | 45 | 133 | 108 | 97 | 31 | 71 | 153 | 127 | 143 | 176 | 171 | 189 | 225 | 218 | 244 | 236 | 234 | 230 | 168 | 177 | 161 | 192 | 197 | 193 | 215 | 254 | 175 | 169 | 296 | 256 | 406 | 469 | 462 | 428 | 1304 | 1310 | 7210 | 7212 |
| Jie1 | 0.110 | 0.101 | 0.127 | 0.117 | 0.108 | - | 7 | 89 | 49 | 130 | 81 | 17 | 52 | 14 | 107 | 80 | 82 | 30 | 69 | 134 | 116 | 132 | 165 | 158 | 177 | 202 | 201 | 223 | 218 | 210 | 207 | 135 | 143 | 127 | 161 | 165 | 159 | 181 | 220 | 145 | 137 | 266 | 221 | 373 | 436 | 430 | 394 | 1316 | 1323 | 7185 | 7180 |
| Nem3 | 0.145 | 0.162 | 0.138 | 0.151 | 0.118 | 0.099 | - | 93 | 51 | 127 | 78 | 13 | 46 | 8 | 101 | 73 | 77 | 31 | 67 | 128 | 112 | 128 | 160 | 153 | 172 | 196 | 195 | 217 | 211 | 204 | 200 | 128 | 137 | 121 | 157 | 161 | 154 | 176 | 213 | 138 | 130 | 259 | 216 | 368 | 431 | 425 | 388 | 1315 | 1322 | 7184 | 7176 |
| Ner1 | 0.107 | 0.139 | 0.119 | 0.086 | 0.112 | 0.072 | 0.119 | - | 47 | 106 | 80 | 87 | 104 | 100 | 183 | 153 | 170 | 116 | 158 | 217 | 205 | 221 | 253 | 246 | 265 | 278 | 284 | 303 | 299 | 284 | 282 | 185 | 187 | 165 | 146 | 156 | 164 | 187 | 253 | 206 | 195 | 323 | 233 | 364 | 421 | 413 | 404 | 1399 | 1406 | 7123 | 7147 |
| Ner2 | 0.084 | 0.101 | 0.109 | 0.138 | 0.108 | 0.089 | 0.097 | 0.095 | - | 93 | 49 | 42 | 58 | 57 | 136 | 106 | 125 | 79 | 117 | 170 | 160 | 176 | 208 | 200 | 219 | 231 | 238 | 256 | 252 | 238 | 235 | 143 | 147 | 126 | 130 | 137 | 138 | 161 | 217 | 161 | 150 | 280 | 206 | 348 | 409 | 402 | 379 | 1365 | 1372 | 7141 | 7147 |
| Jar1 | 0.095 | 0.138 | 0.119 | 0.126 | 0.092 | 0.096 | 0.094 | 0.080 | 0.092 | - | 49 | 114 | 94 | 129 | 158 | 135 | 177 | 159 | 187 | 199 | 209 | 221 | 248 | 237 | 256 | 237 | 260 | 266 | 266 | 239 | 238 | 123 | 118 | 96 | 40 | 51 | 64 | 84 | 162 | 152 | 139 | 249 | 131 | 259 | 318 | 310 | 299 | 1429 | 1436 | 7057 | 7055 |
| Mui1 | 0.098 | 0.116 | 0.123 | 0.138 | 0.106 | 0.103 | 0.102 | 0.100 | 0.077 | 0.077 | - | 65 | 50 | 80 | 125 | 97 | 133 | 110 | 139 | 164 | 167 | 181 | 210 | 200 | 219 | 214 | 229 | 241 | 239 | 218 | 216 | 109 | 110 | 87 | 82 | 88 | 89 | 112 | 173 | 134 | 121 | 246 | 157 | 299 | 360 | 354 | 330 | 1385 | 1392 | 7106 | 7102 |
| Ner3 | 0.104 | 0.108 | 0.111 | 0.126 | 0.122 | 0.073 | 0.075 | 0.068 | 0.075 | 0.077 | 0.070 | - | 36 | 16 | 101 | 71 | 84 | 44 | 78 | 131 | 119 | 135 | 167 | 159 | 178 | 196 | 199 | 219 | 214 | 203 | 200 | 121 | 129 | 112 | 144 | 148 | 142 | 164 | 205 | 133 | 125 | 255 | 205 | 356 | 419 | 413 | 378 | 1327 | 1333 | 7171 | 7164 |
| Nev1 | 0.073 | 0.088 | 0.093 | 0.112 | 0.089 | 0.071 | 0.089 | 0.088 | 0.062 | 0.086 | 0.054 | 0.046 | - | 43 | 81 | 51 | 84 | 73 | 93 | 117 | 117 | 131 | 161 | 151 | 171 | 174 | 184 | 200 | 196 | 180 | 177 | 87 | 94 | 76 | 117 | 119 | 110 | 131 | 169 | 103 | 93 | 223 | 170 | 323 | 387 | 381 | 342 | 1336 | 1343 | 7150 | 7133 |
| Nev2 | 0.069 | 0.134 | 0.096 | 0.120 | 0.093 | 0.098 | 0.130 | 0.108 | 0.090 | 0.100 | 0.098 | 0.085 | 0.070 | - | 94 | 66 | 70 | 30 | 62 | 120 | 105 | 121 | 153 | 146 | 165 | 188 | 188 | 210 | 204 | 196 | 193 | 123 | 132 | 117 | 157 | 160 | 153 | 174 | 209 | 131 | 124 | 253 | 213 | 366 | 430 | 424 | 385 | 1311 | 1317 | 7186 | 7176 |
| Kra1 | 0.116 | 0.145 | 0.146 | 0.115 | 0.078 | 0.103 | 0.101 | 0.106 | 0.111 | 0.072 | 0.067 | 0.067 | 0.078 | 0.094 | - | 30 | 52 | 105 | 87 | 41 | 64 | 70 | 91 | 80 | 98 | 96 | 104 | 120 | 116 | 103 | 100 | 64 | 78 | 81 | 165 | 160 | 141 | 154 | 147 | 51 | 53 | 165 | 175 | 332 | 398 | 394 | 330 | 1276 | 1283 | 7189 | 7143 |
| Dub1 | 0.122 | 0.123 | 0.143 | 0.134 | 0.079 | 0.100 | 0.112 | 0.105 | 0.089 | 0.081 | 0.058 | 0.066 | 0.071 | 0.104 | 0.047 | - | 49 | 82 | 76 | 67 | 75 | 87 | 113 | 103 | 122 | 126 | 133 | 150 | 146 | 132 | 129 | 67 | 79 | 73 | 147 | 144 | 128 | 145 | 155 | 67 | 63 | 188 | 173 | 330 | 396 | 391 | 337 | 1295 | 1302 | 7178 | 7143 |
| Nem4 | 0.109 | 0.119 | 0.086 | 0.109 | 0.060 | 0.080 | 0.093 | 0.088 | 0.107 | 0.052 | 0.089 | 0.073 | 0.078 | 0.078 | 0.075 | 0.074 | - | 66 | 37 | 56 | 35 | 51 | 83 | 76 | 95 | 130 | 121 | 147 | 139 | 140 | 136 | 111 | 125 | 122 | 194 | 192 | 177 | 193 | 198 | 102 | 103 | 213 | 221 | 378 | 444 | 439 | 381 | 1253 | 1260 | 7226 | 7190 |
| Vab1 | 0.132 | 0.123 | 0.137 | 0.119 | 0.098 | 0.093 | 0.100 | 0.093 | 0.097 | 0.098 | 0.073 | 0.063 | 0.089 | 0.105 | 0.070 | 0.054 | 0.066 | - | 43 | 122 | 97 | 113 | 146 | 141 | 159 | 195 | 187 | 213 | 206 | 204 | 200 | 146 | 156 | 143 | 188 | 190 | 183 | 204 | 233 | 149 | 144 | 269 | 241 | 395 | 459 | 453 | 412 | 1287 | 1293 | 7215 | 7206 |
| Ses1 | 0.090 | 0.104 | 0.100 | 0.087 | 0.079 | 0.066 | 0.105 | 0.066 | 0.102 | 0.074 | 0.105 | 0.063 | 0.073 | 0.065 | 0.085 | 0.087 | **0.041** | 0.069 | - | 91 | 58 | 74 | 106 | 102 | 119 | 165 | 151 | 180 | 171 | 175 | 172 | 143 | 155 | 148 | 210 | 210 | 198 | 217 | 231 | 137 | 136 | 250 | 249 | 406 | 471 | 466 | 413 | 1249 | 1256 | 7243 | 7218 |
| Lok1 | 0.128 | 0.102 | 0.117 | 0.151 | 0.104 | 0.092 | 0.086 | 0.098 | 0.076 | 0.072 | 0.097 | 0.081 | 0.083 | 0.108 | 0.111 | 0.085 | 0.080 | 0.082 | 0.068 | - | 42 | 39 | 51 | 40 | 57 | 74 | 68 | 91 | 84 | 84 | 81 | 98 | 112 | 119 | 205 | 200 | 180 | 191 | 171 | 75 | 83 | 165 | 207 | 361 | 427 | 424 | 352 | 1236 | 1243 | 7225 | 7167 |
| Jur1 | 0.136 | 0.127 | 0.130 | 0.119 | 0.081 | 0.111 | 0.135 | 0.112 | 0.140 | 0.109 | 0.119 | 0.085 | 0.117 | 0.139 | 0.101 | 0.078 | 0.062 | 0.055 | 0.088 | 0.106 | - | 16 | 49 | 44 | 62 | 113 | 93 | 123 | 114 | 124 | 120 | 127 | 142 | 143 | 222 | 219 | 202 | 216 | 208 | 110 | 115 | 207 | 239 | 395 | 461 | 458 | 391 | 1220 | 1227 | 7252 | 7205 |
| Vil1 | 0.131 | 0.124 | 0.154 | 0.152 | 0.111 | 0.088 | 0.132 | 0.114 | 0.108 | 0.111 | 0.104 | 0.083 | 0.085 | 0.134 | 0.108 | 0.098 | 0.097 | 0.085 | 0.085 | 0.105 | 0.107 | - | 33 | 28 | 46 | 103 | 79 | 110 | 100 | 114 | 110 | 133 | 147 | 151 | 233 | 229 | 211 | 224 | 210 | 112 | 119 | 201 | 244 | 399 | 465 | 462 | 391 | 1209 | 1216 | 7259 | 7206 |
| Lei1 | 0.091 | 0.120 | 0.120 | 0.111 | 0.098 | 0.105 | 0.142 | 0.078 | 0.083 | 0.094 | 0.105 | 0.088 | 0.094 | 0.082 | 0.115 | 0.085 | 0.085 | 0.085 | 0.060 | 0.070 | 0.104 | 0.109 | - | 12 | 13 | 89 | 53 | 88 | 76 | 100 | 97 | 149 | 162 | 170 | 256 | 251 | 231 | 242 | 217 | 124 | 133 | 192 | 257 | 409 | 475 | 472 | 394 | 1185 | 1192 | 7275 | 7209 |
| Sys1 | 0.096 | 0.127 | 0.137 | 0.096 | 0.103 | 0.082 | 0.131 | 0.082 | 0.114 | 0.085 | 0.096 | 0.074 | 0.075 | 0.102 | 0.093 | 0.093 | 0.095 | 0.095 | 0.082 | 0.088 | 0.108 | 0.088 | 0.070 | - | 20 | 81 | 51 | 84 | 73 | 93 | 89 | 137 | 150 | 159 | 245 | 239 | 220 | 230 | 205 | 112 | 121 | 183 | 245 | 397 | 463 | 460 | 383 | 1196 | 1203 | 7263 | 7199 |
| Atm1 | 0.115 | 0.198 | 0.175 | 0.147 | 0.116 | 0.137 | 0.091 | 0.115 | 0.121 | 0.073 | 0.121 | 0.103 | 0.115 | 0.125 | 0.093 | 0.096 | 0.117 | 0.112 | 0.129 | 0.127 | 0.151 | 0.125 | 0.136 | 0.129 | - | 81 | 42 | 77 | 65 | 93 | 89 | 152 | 166 | 175 | 263 | 257 | 236 | 246 | 216 | 126 | 136 | 185 | 258 | 409 | 474 | 472 | 391 | 1179 | 1186 | 7276 | 7206 |
| Sal1 | 0.133 | 0.123 | 0.189 | 0.191 | 0.133 | 0.147 | 0.197 | 0.134 | 0.155 | 0.130 | 0.138 | 0.136 | 0.107 | 0.176 | 0.147 | 0.132 | 0.133 | 0.147 | 0.132 | 0.150 | 0.143 | 0.096 | 0.139 | 0.127 | 0.170 | - | 46 | 30 | 35 | 12 | 8 | 115 | 124 | 143 | 231 | 222 | 199 | 201 | 150 | 85 | 98 | 104 | 200 | 341 | 405 | 404 | 314 | 1229 | 1236 | 7211 | 7127 |
| Dan1 | 0.122 | 0.128 | 0.161 | 0.149 | 0.128 | 0.139 | 0.148 | 0.122 | 0.145 | 0.132 | 0.119 | 0.111 | 0.127 | 0.165 | 0.121 | 0.110 | 0.114 | 0.109 | 0.104 | 0.135 | 0.109 | 0.118 | 0.151 | 0.142 | 0.157 | 0.111 | - | 35 | 23 | 56 | 53 | 144 | 156 | 170 | 260 | 252 | 230 | 236 | 193 | 115 | 127 | 148 | 240 | 385 | 449 | 448 | 360 | 1190 | 1197 | 7255 | 7172 |
| Sve1 | 0.067 | 0.077 | 0.133 | 0.120 | 0.090 | 0.087 | 0.114 | 0.085 | 0.068 | 0.073 | 0.086 | 0.079 | 0.069 | 0.104 | 0.102 | 0.077 | 0.079 | 0.094 | 0.075 | 0.076 | 0.093 | 0.106 | 0.082 | 0.101 | 0.115 | 0.083 | 0.078 | - | 13 | 33 | 32 | 145 | 154 | 172 | 261 | 252 | 229 | 231 | 177 | 115 | 128 | 118 | 229 | 366 | 429 | 428 | 335 | 1201 | 1209 | 7236 | 7143 |
| Raz1 | 0.117 | 0.126 | 0.147 | 0.157 | 0.085 | 0.140 | 0.142 | 0.145 | 0.131 | 0.117 | 0.136 | 0.119 | 0.111 | 0.136 | 0.123 | 0.122 | 0.096 | 0.130 | 0.117 | 0.117 | 0.117 | 0.133 | 0.137 | 0.140 | 0.155 | 0.123 | 0.088 | 0.061 | - | 42 | 40 | 147 | 157 | 174 | 263 | 255 | 232 | 235 | 185 | 117 | 130 | 130 | 235 | 375 | 439 | 438 | 346 | 1194 | 1201 | 7245 | 7155 |
| Bar1 | 0.112 | 0.127 | 0.127 | 0.165 | 0.115 | 0.152 | 0.147 | 0.132 | 0.094 | 0.100 | 0.092 | 0.112 | 0.098 | 0.106 | 0.116 | 0.106 | 0.130 | 0.158 | 0.138 | 0.131 | 0.161 | 0.146 | 0.129 | 0.146 | 0.133 | 0.109 | 0.126 | 0.070 | 0.102 | - | **4** | 116 | 124 | 144 | 231 | 222 | 198 | 199 | 144 | 87 | 100 | 93 | 196 | 333 | 397 | 396 | 305 | 1234 | 1242 | 7204 | 7117 |
| Erl1 | 0.104 | 0.121 | 0.138 | 0.145 | 0.117 | 0.148 | 0.171 | 0.127 | 0.139 | 0.108 | 0.123 | 0.114 | 0.106 | 0.125 | 0.097 | 0.128 | 0.101 | 0.159 | 0.104 | 0.149 | 0.150 | 0.157 | 0.161 | 0.160 | 0.158 | 0.111 | 0.125 | 0.073 | 0.097 | 0.089 | - | 115 | 123 | 142 | 230 | 221 | 198 | 199 | 145 | 85 | 98 | 96 | 196 | 335 | 399 | 398 | 308 | 1233 | 1241 | 7206 | 7120 |
| Kul1 | 0.156 | 0.158 | 0.210 | 0.156 | 0.133 | 0.152 | 0.158 | 0.140 | 0.153 | 0.183 | 0.156 | 0.168 | 0.155 | 0.189 | 0.160 | 0.164 | 0.148 | 0.163 | 0.140 | 0.146 | 0.137 | 0.165 | 0.167 | 0.163 | 0.190 | 0.143 | 0.140 | 0.112 | 0.173 | 0.167 | 0.137 | - | 14 | 28 | 116 | 108 | 86 | 94 | 89 | 30 | 17 | 138 | 111 | 268 | 334 | 330 | 271 | 1331 | 1338 | 7127 | 7080 |
| Mus1 | 0.150 | 0.191 | 0.224 | 0.152 | 0.169 | 0.186 | 0.162 | 0.176 | 0.165 | 0.144 | 0.145 | 0.123 | 0.146 | 0.168 | 0.125 | 0.115 | 0.153 | 0.137 | 0.171 | 0.175 | 0.152 | 0.195 | 0.177 | 0.171 | 0.125 | 0.185 | 0.137 | 0.088 | 0.110 | 0.118 | 0.140 | 0.209 | - | 23 | 107 | 98 | 75 | 81 | 77 | 41 | 30 | 136 | 97 | 254 | 320 | 316 | 258 | 1344 | 1351 | 7113 | 7066 |
| Kru1 | 0.114 | 0.130 | 0.141 | 0.123 | 0.091 | 0.139 | 0.139 | 0.131 | 0.112 | 0.133 | 0.118 | 0.101 | 0.100 | 0.133 | 0.117 | 0.114 | 0.094 | 0.095 | 0.114 | 0.131 | 0.099 | 0.118 | 0.141 | 0.147 | 0.123 | 0.107 | 0.080 | 0.059 | 0.062 | 0.101 | 0.099 | 0.134 | 0.091 | - | 90 | 82 | 61 | 73 | 93 | 58 | 44 | 159 | 100 | 257 | 323 | 318 | 268 | 1354 | 1362 | 7108 | 7070 |
| Nee1 | 0.107 | 0.126 | 0.152 | 0.124 | 0.096 | 0.114 | 0.138 | 0.100 | 0.105 | 0.115 | 0.085 | 0.085 | 0.091 | 0.131 | 0.108 | 0.072 | 0.109 | 0.098 | 0.109 | 0.117 | 0.087 | 0.118 | 0.108 | 0.099 | 0.152 | 0.097 | 0.077 | 0.046 | 0.082 | 0.078 | 0.119 | 0.112 | 0.091 | 0.075 | - | 13 | 35 | 49 | 133 | 147 | 134 | 226 | 94 | 219 | 279 | 272 | 259 | 1441 | 1448 | 7033 | 7020 |
| Nee2 | 0.124 | 0.141 | 0.161 | 0.136 | 0.094 | 0.157 | 0.159 | 0.137 | 0.172 | 0.124 | 0.136 | 0.120 | 0.137 | 0.147 | 0.111 | 0.117 | 0.089 | 0.116 | 0.102 | 0.140 | 0.075 | 0.153 | 0.144 | 0.159 | 0.159 | 0.125 | 0.102 | 0.069 | 0.089 | 0.123 | 0.094 | 0.124 | 0.097 | 0.082 | 0.077 | - | 24 | 36 | 119 | 138 | 125 | 214 | 81 | 211 | 273 | 266 | 248 | 1436 | 1443 | 7034 | 7016 |
| Apa1 | 0.102 | 0.092 | 0.093 | 0.166 | 0.147 | 0.134 | 0.136 | 0.120 | 0.088 | 0.118 | 0.086 | 0.079 | 0.102 | 0.106 | 0.118 | 0.110 | 0.123 | 0.129 | 0.140 | 0.104 | 0.124 | 0.157 | 0.131 | 0.143 | 0.149 | 0.155 | 0.135 | 0.086 | 0.139 | 0.090 | 0.099 | 0.162 | 0.121 | 0.110 | 0.111 | 0.109 | - | 23 | 99 | 116 | 103 | 191 | 70 | 214 | 277 | 271 | 242 | 1416 | 1423 | 7050 | 7023 |
| Apa2 | 0.111 | 0.137 | 0.137 | 0.179 | 0.127 | 0.132 | 0.136 | 0.127 | 0.110 | 0.123 | 0.110 | 0.080 | 0.103 | 0.121 | 0.119 | 0.086 | 0.114 | 0.085 | 0.110 | 0.093 | 0.103 | 0.137 | 0.122 | 0.127 | 0.156 | 0.150 | 0.083 | 0.079 | 0.088 | 0.118 | 0.142 | 0.169 | 0.104 | 0.110 | 0.063 | 0.110 | 0.102 | - | 85 | 122 | 110 | 181 | 47 | 192 | 256 | 250 | 218 | 1424 | 1432 | 7035 | 7003 |
| Lie1 | 0.127 | 0.092 | 0.108 | 0.191 | 0.110 | 0.123 | 0.116 | 0.141 | 0.102 | 0.089 | 0.084 | 0.066 | 0.082 | 0.134 | 0.112 | 0.090 | 0.092 | 0.119 | 0.117 | 0.090 | 0.098 | 0.120 | 0.128 | 0.136 | 0.138 | 0.141 | 0.138 | 0.073 | 0.099 | 0.087 | 0.104 | 0.169 | 0.121 | 0.100 | 0.078 | 0.106 | 0.071 | 0.085 | - | 98 | 95 | 100 | 57 | 193 | 258 | 256 | 183 | 1378 | 1385 | 7062 | 6996 |
| Ven1 | 0.082 | 0.100 | 0.115 | 0.137 | 0.090 | 0.095 | 0.134 | 0.103 | 0.090 | 0.094 | 0.087 | 0.076 | 0.071 | 0.084 | 0.079 | 0.077 | 0.085 | 0.106 | 0.096 | 0.119 | 0.100 | 0.108 | 0.132 | 0.128 | 0.127 | 0.095 | 0.095 | 0.055 | 0.090 | 0.058 | 0.050 | 0.133 | 0.112 | 0.086 | 0.071 | 0.091 | 0.067 | 0.076 | 0.074 | - | 13 | 122 | 133 | 287 | 353 | 350 | 281 | 1303 | 1310 | 7151 | 7094 |
| Rin1 | 0.144 | 0.169 | 0.162 | 0.189 | 0.125 | 0.148 | 0.189 | 0.129 | 0.137 | 0.162 | 0.145 | 0.125 | 0.124 | 0.167 | 0.153 | 0.123 | 0.132 | 0.124 | 0.137 | 0.143 | 0.103 | 0.153 | 0.117 | 0.156 | 0.213 | 0.180 | 0.151 | 0.100 | 0.138 | 0.152 | 0.163 | 0.178 | 0.175 | 0.151 | 0.110 | 0.128 | 0.160 | 0.091 | 0.121 | 0.116 | - | 130 | 125 | 280 | 346 | 343 | 278 | 1314 | 1321 | 7142 | 7090 |
| Ven2 | 0.122 | 0.118 | 0.143 | 0.122 | 0.091 | 0.113 | 0.160 | 0.110 | 0.152 | 0.129 | 0.103 | 0.106 | 0.116 | 0.132 | 0.089 | 0.102 | 0.082 | 0.090 | 0.097 | 0.155 | 0.083 | 0.148 | 0.152 | 0.130 | 0.164 | 0.138 | 0.088 | 0.078 | 0.095 | 0.141 | 0.086 | 0.115 | 0.126 | 0.079 | 0.076 | 0.066 | 0.115 | 0.114 | 0.106 | 0.079 | 0.127 | - | 156 | 262 | 322 | 323 | 220 | 1300 | 1307 | 7126 | 7026 |
| Kau1 | 0.141 | 0.123 | 0.156 | 0.166 | 0.118 | 0.161 | 0.172 | 0.153 | 0.124 | 0.166 | 0.119 | 0.125 | 0.111 | 0.151 | 0.123 | 0.106 | 0.121 | 0.108 | 0.130 | 0.128 | 0.127 | 0.144 | 0.146 | 0.162 | 0.184 | 0.113 | 0.120 | 0.069 | 0.098 | 0.101 | 0.078 | 0.123 | 0.124 | 0.064 | 0.081 | 0.119 | 0.120 | 0.106 | 0.106 | 0.075 | 0.127 | 0.099 | - | 157 | 223 | 219 | 174 | 1429 | 1437 | 7018 | 6970 |
| Ohn1 | 0.151 | 0.097 | 0.146 | 0.152 | 0.136 | 0.144 | 0.185 | 0.136 | 0.151 | 0.152 | 0.125 | 0.132 | 0.124 | 0.191 | 0.157 | 0.123 | 0.132 | 0.140 | 0.143 | 0.156 | 0.113 | 0.142 | 0.177 | 0.175 | 0.166 | 0.141 | 0.095 | 0.086 | 0.100 | 0.133 | 0.115 | 0.160 | 0.109 | 0.086 | 0.081 | 0.100 | 0.114 | 0.113 | 0.080 | 0.093 | 0.135 | 0.089 | 0.098 | - | 66 | 63 | 87 | 1562 | 1569 | 6871 | 6813 |
| Mud1 | 0.126 | 0.087 | 0.133 | 0.139 | 0.124 | 0.130 | 0.184 | 0.129 | 0.128 | 0.160 | 0.110 | 0.106 | 0.110 | 0.133 | 0.128 | 0.097 | 0.126 | 0.120 | 0.125 | 0.134 | 0.091 | 0.144 | 0.144 | 0.161 | 0.191 | 0.133 | 0.100 | 0.062 | 0.096 | 0.098 | 0.088 | 0.139 | 0.107 | 0.076 | 0.078 | 0.068 | 0.061 | 0.095 | 0.090 | 0.056 | 0.119 | 0.071 | 0.078 | 0.069 | - | 11 | 118 | 1622 | 1629 | 6807 | 6748 |
| Ema1 | 0.092 | 0.140 | 0.118 | 0.135 | 0.096 | 0.131 | 0.148 | 0.140 | 0.118 | 0.110 | 0.103 | 0.123 | 0.110 | 0.114 | 0.132 | 0.133 | 0.082 | 0.141 | 0.113 | 0.140 | 0.117 | 0.147 | 0.147 | 0.158 | 0.185 | 0.158 | 0.093 | 0.057 | 0.067 | 0.088 | 0.094 | 0.153 | 0.113 | 0.081 | 0.067 | 0.093 | 0.120 | 0.095 | 0.089 | 0.075 | 0.118 | 0.081 | 0.087 | 0.102 | 0.088 | - | 124 | 1623 | 1630 | 6808 | 6753 |
| Rei1 | 0.114 | 0.121 | 0.143 | 0.100 | 0.109 | 0.100 | 0.159 | 0.084 | 0.105 | 0.115 | 0.087 | 0.111 | 0.108 | 0.132 | 0.103 | 0.109 | 0.095 | 0.101 | 0.098 | 0.126 | 0.089 | 0.137 | 0.126 | 0.141 | 0.178 | 0.139 | 0.122 | 0.061 | 0.114 | 0.094 | 0.086 | 0.122 | 0.137 | 0.092 | 0.078 | 0.092 | 0.104 | 0.129 | 0.111 | 0.065 | 0.121 | 0.067 | 0.085 | 0.107 | 0.063 | 0.070 | - | 1513 | 1520 | 6908 | 6816 |
| Our1 | 0.131 | 0.178 | 0.183 | 0.144 | 0.156 | 0.174 | 0.223 | 0.151 | 0.185 | 0.195 | 0.156 | 0.144 | 0.124 | 0.175 | 0.165 | 0.145 | 0.161 | 0.173 | 0.150 | 0.193 | 0.141 | 0.190 | 0.179 | 0.175 | 0.217 | 0.157 | 0.107 | 0.104 | 0.122 | 0.138 | 0.126 | 0.174 | 0.118 | 0.104 | 0.089 | 0.125 | 0.155 | 0.116 | 0.135 | 0.107 | 0.105 | 0.105 | 0.100 | 0.083 | 0.092 | 0.105 | 0.107 | - | 8 | 8408 | 8134 |
| Sau1 | 0.118 | 0.146 | 0.148 | 0.133 | 0.123 | 0.145 | 0.149 | 0.140 | 0.107 | 0.142 | 0.083 | 0.125 | 0.101 | 0.129 | 0.121 | 0.119 | 0.127 | 0.134 | 0.156 | 0.154 | 0.143 | 0.171 | 0.155 | 0.169 | 0.176 | 0.172 | 0.100 | 0.078 | 0.085 | 0.073 | 0.113 | 0.165 | 0.075 | 0.082 | 0.076 | 0.118 | 0.104 | 0.092 | 0.108 | 0.078 | 0.109 | 0.106 | 0.089 | 0.092 | 0.081 | 0.044 | 0.083 | 0.087 | - | **8416** | 8142 |
| Yas1 | 0.274 | 0.235 | 0.260 | 0.297 | 0.265 | 0.262 | 0.302 | 0.213 | 0.282 | 0.260 | 0.280 | 0.219 | 0.260 | 0.258 | 0.228 | 0.235 | 0.255 | 0.241 | 0.248 | 0.286 | 0.250 | 0.280 | 0.256 | 0.249 | 0.270 | 0.234 | 0.192 | 0.227 | 0.179 | 0.216 | 0.257 | 0.280 | 0.255 | 0.222 | 0.193 | 0.223 | 0.252 | 0.175 | 0.230 | 0.223 | 0.281 | 0.186 | 0.280 | 0.229 | 0.211 | 0.263 | 0.251 | 0.252 | 0.259 | - | 2275 |
| Kir1 | 0.231 | 0.223 | 0.227 | 0.249 | 0.229 | 0.267 | **0.320** | 0.210 | 0.246 | 0.256 | 0.282 | 0.250 | 0.258 | 0.229 | 0.242 | 0.267 | 0.243 | 0.258 | 0.210 | 0.265 | 0.277 | 0.245 | 0.224 | 0.224 | 0.250 | 0.241 | 0.209 | 0.203 | 0.184 | 0.187 | 0.222 | 0.279 | 0.240 | 0.193 | 0.215 | 0.242 | 0.268 | 0.230 | 0.253 | 0.232 | 0.251 | 0.198 | 0.210 | 0.221 | 0.218 | 0.206 | 0.225 | 0.251 | 0.212 | 0.198 | - |

Population codes: Nem1 – Nemunas (Druskininkai, Nemunas basin, LT), Mer1 – Merkys (Dargužiai, Nemunas basin, LT), Mer2 – Merkys (Perloja, Nemunas basin, LT), Mer3 – Merkys (Merkinė, Nemunas basin, LT), Nem2 – Nemunas (Balbieriškis, Nemunas basin, LT), Jie1 – Jiesia (Rokai, Nemunas basin, LT), Ner1 – Neris (Naujieji Verkiai, Nemunas basin, LT), Ner2 – Neris (Čiobiškis, Nemunas basin, LT), Jar1 – Jara (Svėdasai, Nemunas basin, LT), Mui1 – Mušia (Taujėnai, Nemunas basin, LT), Ner3 – Neris (Lapės, Nemunas basin, LT), Nev1 – Nevėžis (Kėdainiai, Nemunas basin, LT), Nev2 – Nevėžis (Raudondvaris, Nemunas basin, LT), Kra1 – Kražantė (Kelmė, Nemunas basin, LT), Dub1 – Dubysa (Daugodai, Nemunas basin, LT), Nem4 – Nemunas (Jurbarkas, Nemunas basin, LT), Vab1 – Vabalkšnė (Ąžuolų Būda, Nemunas basin, LT), Ses1 – Šešupė (Kudirkos Naumiestis, Nemunas basin, LT), Lok1 – Lokysta (Šilalė, Nemunas basin, LT), Jus1 – Jūra (Mociškiai, Nemunas basin, LT), Vil1 – Vilka (Gudai, Nemunas basin, LT), Lie1 – Leitė (Sausgalviai, Nemunas basin, LT), Sys1 – Šyša (Rumšai, Nemunas basin, LT), Atm1 – Atmata (Uostadvaris, Nemunas basin, LT), Sal1 – Salandas (Salantai, Coastal rivers basin, LT), Dan1 – Danė (Klaipėda, Coastal rivers basin, LT), Sve1 – Šventoji (Šventoji, Coastal rivers basin, LT) Raz1 – Rąžė

(Palanga, Coastal rivers basin, LT), Bar1 – Bartuva (Mosėdis, Bartuva basin, LT), Erl1 – Erla (Šaukliai, Bartuva basin, LT), Kul1 – Kulpė (Jurgaičiai, Lielupe basin, LT), Mus1 – Mūša (Mekiai, Lielupe basin, LT), Kru1 – Kruoja (Pakruojis, Lielupe basin, LT), Nee1 – Nemunėlis (Panemunėlis, Lielupe basin, LT), Nee2 – Nemunėlis (Kvetkai, Lielupe basin, LT), Apa1 – Apaščia (Biržai, Lielupe basin, LT), Apa2 – Apaščia (Nemunėlio Radviliškis, Lielupe basin, LT), Lie1 – Lielupe (Kalnciemas, Lielupe basin, LV), Ven1 – Venta (Kuršėnai, Venta basin, LT), Rin1 – Ringuva (Kužiai, Venta basin, LT), Ven2 – Venta (Kuldyga Venta basin, LV), Kau1 – Kausupite (Berkava, Dauguva basin, LV), Ohn1 – Ohne (Torva, Vorstjarv basin, EE), Mud1 – Muda (Sojamma, Peipus basin, EE), Ema1 – Emajogi (Tartu, Peipus basin, EE), Rei1 – Reiu (Parnu, Parnu basin, EE), Our1 – Our (Vianden, Rein basin, LU), Sau1 – Sauer (Diekrich, Rein basin, LU), Yas1 – Ob'Yasneniya (Vladivastok, Japan sea basin, RU), Kir1 – Kirpichnava (Petropavlovsk-Kamchaskiy, Khalaktyrskoye basin, RU).

**Table S3.** Geographical locations of Eurasian *Phalaris arundinacea* populations used for genetic analysis: population code, DNA marker type, river name, river basin name, site name, country of origin, and geographical location specifics (latitude, longitude, altitude).

| Population | Title in GBS-SNP analysis | DNA marker type* | River | River basin | Site | Country | Geographical location | | |
|---|---|---|---|---|---|---|---|---|---|
| | | | | | | | Latitude (°N) | Longitude (°E) | Altitude (m) |
| Nem1 | LT3 | a,b | Nemunas | Nemunas | Druskininkai | LT | 54°01'12.5" | 23°58'53.5" | 81 |
| Ber | LT1 | b | Beržė | Merkys | Road 3903 | LT | 54º17′51.05" | 25º12′40.71" | 141 |
| Mer1 | | a | Merkys | Nemunas | Dargužiai | LT | 54°22'29.1" | 24°52'51.6" | 119 |
| Mer2 | | a | Merkys | Nemunas | Perloja | LT | 54°12'43.9" | 24°25'12.2" | 98 |
| Mer3 | | a | Merkys | Nemunas | Merkinė | LT | 54°09'26.8" | 24°10'56.9" | 74 |
| Mer4 | LT2 | b | Merkys | Merkys | Piramidė Road 4 | LT | 54º08′38.20" | 24º12′38.40" | 72 |
| Nem2 | | a | Nemunas | Nemunas | Balbieriškis | LT | 54°31'49.8" | 23°53'15.9" | 48 |
| Jie1 | | a | Jiesia | Nemunas | Rokai | LT | 54°50'15.7" | 23°56'14.3" | 34 |
| Nem3 | LT4 | a,b | Nemunas | Nemunas | Kaunas | LT | 54°53'35.9" | 23°53'21.0" | 17 |
| Ner1 | | a | Neris | Nemunas | Naujieji Verkiai | LT | 54°44'58.9" | 25°19'16.1" | 99 |
| Ner2 | LT5 | a,b | Neris | Nemunas | Čiobiškis | LT | 54°56'55.9" | 24°40'28.0" | 49 |
| Jar1 | | a | Jara | Nemunas | Svėdasai | LT | 55°42'08.6" | 25°18'45.8" | 83 |
| Mui1 | | a | Mūšia | Nemunas | Taujėnai | LT | 55°23'24.2" | 24°45'51.1" | 67 |
| Ner3 | | a | Neris | Nemunas | Lapės | LT | 54°58'46.2" | 24°01'37.7" | 34 |
| Nev1 | | a | Nevėžis | Nemunas | Kėdainiai | LT | 55°17'58.3" | 23°59'46.3" | 31 |
| Nev2 | | a | Nevėžis | Nemunas | Raudondvaris | LT | 54°55'46.4" | 23°47'26.0" | 21 |
| Kra1 | | a | Kražantė | Nemunas | Kelmė | LT | 55°33'19.0" | 22°48'12.2" | 128 |
| Dub1 | | a | Dubysa | Nemunas | Daugodai | LT | 55°25'31.9" | 23°13'30.1" | 56 |
| Nem4 | | a | Nemunas | Nemunas | Jurbarkas | LT | 55°05'35.1" | 22°43'48.9" | 19 |
| Vab1 | | a | Vabalkšnė | Nemunas | Ąžuolų Būda | LT | 54°42'22.8" | 23°31'20.2" | 54 |
| Ses1 | | a | Šešupė | Nemunas | Kudrikos Naumiestis | LT | 54°46'16.4" | 22°52'0.22" | 36 |
| Lok1 | | a | Lokysta | Nemunas | Šilalė | LT | 55°29'12.5" | 22°10'06.1" | 91 |
| Jur1 | | a | Jūra | Nemunas | Mociškiai | LT | 55°06'29.3" | 22°10'43.5" | 6 |
| Vil1 | | a | Vilka | Nemunas | Gudai | LT | 55°09'46.4" | 21°56'22.5" | 5 |
| Lei1 | | a | Leitė | Nemunas | Sausgalviai | LT | 55°15'57.6" | 21°27'18.4" | 1 |
| Sys1 | | a | Šyša | Nemunas | Rumšai | LT | 55°19'54.5" | 21°36'13.3" | 7 |
| Atm1 | LT6 | a,b | Atmata | Nemunas | Uostadvaris | LT | 55°20'42.5" | 21°17'42.8" | 1 |
| Sal1 | | a | Salantas | Seaside rivers | Salantai | LT | 56°03'35.8" | 21°33'58.3" | 42 |
| Dan1 | | a | Danė | Seaside rivers | Klaipėda | LT | 55°43'03.6" | 21°10'3.79" | 2 |
| Sve1 | | a | Šventoji | Seaside rivers | Šventoji | LT | 56°01'51.6" | 21°05'14.6" | 1 |
| Raz1 | | a | Rąžė | Seaside rivers | Palanga | LT | 55°54'59.3" | 21°03'56.4" | 5 |
| Bar1 | | a | Bartuva | Bartuva | Mosėdis | LT | 56°09'54.2" | 21°33'51.8" | 37 |

| | | | | | | | | |
|---|---|---|---|---|---|---|---|---|
| Erl1 | a | Erla | Bartuva | Šaukliai | LT | 56°07'51.1" | 21°34'43.0" | 48 |
| Kul1 | a | Kulpė | Lielupe | Jurgaičiai | LT | 56°00'51.8" | 23°24'57.0" | 95 |
| Mus1 | a | Mūša | Lielupe | Mekiai | LT | 56°06'34.9" | 23°34'03.2" | 71 |
| Kru1 | a | Kruoja | Lielupe | Pakruojis | LT | 55°58'52.4" | 23°51'15.7" | 59 |
| Nee1 | a | Nemunėlis | Lielupe | Panemunėlis | LT | 56°03'42.8" | 25°17'15.0" | 84 |
| Nee2 | a | Nemunėlis | Lielupe | Kvetkai | LT | 56°09'03.6" | 25°08'27.5" | 72 |
| Apa1 | a | Apaščia | Lielupe | Biržai | LT | 56°11'16.7" | 24°45'57.8" | 56 |
| Apa2 | a | Apaščia | Lielupe | Nemunėlio Radviliškis | LT | 56°23'51.5" | 24°46'02.0" | 38 |
| Ven1 | a | Venta | Venta | Kuršėnai | LT | 56°00'16.3" | 22°55'49.7" | 97 |
| Rin1 | a | Ringuva | Venta | Kužiai | LT | 55°59'12.4" | 23°08'35.2" | 101 |
| Lie1 | a | Lielupė | Lielupe | Kalnciemas | LV | 56°48'15.6" | 23°36'03.9" | 1 |
| Ven2 | a | Venta | Venta | Kuldyga | LV | 56°57'58.9" | 21°58'59.3" | 24 |
| Kau1 | a | Kausupite | Dauguva | Berkava | LV | 56°48'01.4" | 24°32'17.7" | 22 |
| Ohn1 | a | Ohne | Vortsjarv | Torva | EE | 58°00'11.9" | 25°55'14.7" | 52 |
| Mud1 | a | Muda | Peipus | Sojamma | EE | 58°28'01.6" | 26°37'34.8" | 39 |
| Ema1 | a | Emajogi | Peipus | Tartu | EE | 58°23'00.1" | 26°43'28.7" | 40 |
| Rei1 | a | Reiu | Pernu | Parnu | EE | 58°21'36.8" | 24°36'16.7" | 6 |
| Our1 | a | Our | Rein | Vianden | LU | 49°56'01.6" | 6°12'23.40" | 203 |
| Sau1 | a | Sauer | Rein | Diekirch | LU | 49°51'57.9" | 6°09'37.00" | 189 |
| Yas1 | a | Ob'Yasneniya | Ob'Yasneniya | Vladivastok | RU | 43°06'08.9" | 131°56'40.7" | 7 |
| Kir1 | a | Kirpichnaya | Khalaktyrskoye | Petropavlovsk-Kamchatskiy | RU | 54°18'17.8" | 158°13'05.8" | 255 |

Country: LT – Lithuania, LV – Latvia, EE – Estonia, LU – Luxembourg, RU – Russian Far East. *DNA marker type: a – microsatellite, b – GBS (genotype by sequencing) to generate single nucleotide polymorphisms (SNPs).

**Table S4.** Site information (population numerical codes, geographic locations of latitude and longitude, and altitude) of 45 Lithuanian genotype accessions of ornamental *Phalaris arundinacea* var. *picta* or *P. arundinacea*

| Population | Geographic location | | |
|---|---|---|---|
| | Latitude (°N) | Longitude (°E) | Altitude (m) |
| Druskininkai 1 | 54°01'14.8" | 23°58'30.7" | 96 |
| Druskininkai 2 | 54°00'45.2" | 23°58'57.7" | 96 |
| Taujėnai | 55°23'45.9" | 24°45'37.8" | 79 |
| Lazdijai | 54°14'01.0" | 23°31'05.9" | 122 |
| Simnas | 54°23'01.2" | 24°39'16.9" | 133 |
| Rumokai | 54º43'22.0" | 22º58'59.9" | 51 |
| Kaunas 1 | 54º52'11.2" | 23º54'36.1" | 75 |
| Kaunas 2 | 54º52'30.0" | 23º56'06.9" | 30 |
| Kaunas 3 | 54°52'35.0" | 23°56'22.7" | 41 |
| Kaunas 4 | 55º53'23.2" | 23º56'40.9" | 65 |
| Kaunas 5 | 54°52'28.1" | 23°56'08.7" | 29 |
| Kaunas 6 | 54º54'18.8" | 23º56'25.7" | 73 |
| Kaunas 7 | 54º55'21.4" | 23º56'15.9" | 74 |
| Kaunas 8 | 54°55'18.5" | 23°56'34.8" | 76 |
| Kaunas 9 | 54°54'16.3" | 23°58'22.3" | 65 |
| Muniškiai | 55°01'20.0" | 23°48'20.2" | 56 |
| Vaišvydava | 54°50'28.0" | 24°02'44.9" | 78 |
| Laumėnai | 54°52'23.1" | 24°03'42.1" | 64 |
| Rumšiškės | 54°52'01.7" | 24°12'37.7" | 67 |
| Vilnius | 54°41'56.1" | 25°15'16.4" | 108 |
| Drabužininkai | 54°34'41.7" | 24°38'37.4" | 137 |

| | | | |
|---|---|---|---|
| Onuškis | 54°29'03.9" | 24°35'39.0" | 142 |
| Širvintos | 55°02'13.3" | 24°58'01.3" | 119 |
| Ukmergė | 55°14'32.7" | 24°46'24.9" | 59 |
| Utena | 55°29'46.1" | 25°35'14.2" | 111 |
| Naujas Janavas | 55°02'55.6" | 26°13'42.1" | 206 |
| Dotnuva | 55°23'40.3" | 23°51'28.5" | 71 |
| Jošvainiai | 55°14'47.9" | 23°50'27.4" | 49 |
| Ariogala | 55°15'49.0" | 23°28'14.6" | 82 |
| Germanikis | 56°22'27.7" | 24°39'19.6" | 39 |
| Kirkilai | 56°14'31.5" | 24°41'33.8" | 46 |
| Žagarė 1 | 56°21'17.1" | 23°15'30.1" | 70 |
| Žagarė 2 | 56°21'28.7" | 23°15'22.9" | 65 |
| Kuršėnai | 56°00'19.8" | 22°55'48.7" | 103 |
| Lumpėnai | 55°06'26.8" | 22°02'17.7" | 26 |
| Nemakščiai | 55°25'51.9" | 22°46'09.8" | 108 |
| Šilalė | 55°29'18.5" | 22°10'39.4" | 96 |
| Mosėdis | 56°10'01.1" | 21°34'38.9" | 50 |
| Baubliai | 55°49'46.6" | 21°24'21.3" | 61 |
| Palanga 1 | 55°55'16.0" | 21°04'18.7" | 6 |
| Palanga 2 | 55°55'06.2" | 21°03'48.5" | 3 |
| Palanga 3 | 55°54'34.1" | 21°04'05.5" | 10 |
| Palanga 4 | 55°55'12.4" | 21°03'41.2" | 4 |
| Palanga 5 | 55°55'17.7" | 21°03'43.1" | 4 |
| Telšiai | 55°58'59.0" | 22°15'47.4" | 132 |

**Table S5**. Characteristics of the environment factors for Lithuanian *Phalaris arundinacea* populations used for genetical analysis: population, land cover type, river state, geographica areas based on N concentration in years 1992–1996, river size, and riverbed origin.

| Population | Land cover type | River state | Geographic areas based on N concentrations in years 1992–1996 | River size | Riverbed origin |
|---|---|---|---|---|---|
| Nem1 | AGR | MO | SE | XL | N |
| Mer1 | FOR | G | SE | L | N |
| Mer2 | ART | G | SE | L | N |
| Mer3 | AGR | G | SE | L | N |
| Nem2 | AGR | MO | SE | XL | N |
| Jie1 | ART | MO | C | M | N |
| Nem3 | ART | MO | C | XL | N |
| Ner1 | ART | MO | SE | XL | N |
| Ner2 | AGR | MO | SE | XL | N |
| Jar1 | AGR | G | SE | M | N |
| Mui1 | AGR | B | SE | M | R |
| Ner3 | AGR | MO | C | XL | N |
| Nev1 | AGR | P | C | L | N |
| Nev2 | FOR | MO | C | L | N |
| Kra1 | FOR | G | C | M | N |
| Dub1 | AGR | G | C | L | N |
| Nem4 | AGR | MO | C | XL | N |
| Vab1 | AGR | G | C | M | N |
| Ses1 | ART | G | C | L | N |

| | | | | | |
|---|---|---|---|---|---|
| Lok1 | AGR | G | NW | M | N |
| Jur1 | AGR | G | NW | L | N |
| Vil1 | AGR | MO | NW | M | R |
| Lei1 | FOR | MO | NW | M | R |
| Sys1 | AGR | G | NW | M | N |
| Atm1 | AGR | P | NW | M | N |
| Sal1 | ART | G | NW | M | N |
| Dan1 | ART | MO | NW | M | N |
| Sve1 | ART | MO | NW | M | N |
| Raz1 | ART | B | NW | S | R |
| Bar1 | AGR | P | NW | M | N |
| Erl1 | AGR | B | NW | M | N |
| Kul1 | AGR | MO | C | M | N |
| Mus1 | AGR | P | C | L | R |
| Kru1 | ART | P | C | M | N |
| Nee1 | ART | MO | C | L | N |
| Nee2 | ART | MO | C | L | N |
| Apa1 | ART | G | C | M | N |
| Apa2 | ART | G | C | M | N |
| Ven1 | ART | B | C | XL | N |
| Rin1 | ART | MO | C | M | R |

Land cover types: AGR – agricultural surfaces, ART – artificial surfaces, FOR – forest and semi-natural areas; river state: H – high, G – good, MO – moderate, P – poor, B – bad; geographic areas based on N concentrations: SE – South-East of Lithuania, C – central Lithuania, NW – North-West of Lithuania; river size: XL – extra large (>10000 $km^2$), L – large (1000–10000 $km^2$), M – medium (100–1000 $km^2$), S – small (<100 $km^2$); riverbed origin: N – natural, R – regulated.
